# Supplementary material for: AETA peptide contributes to Alzheimer’s disease signature of synapse dysfunction
Source: Acta Neuropathol. 2026 Jun 3;151(1):65. doi: 10.1007/s00401-026-03033-2 (PMC13233929; doi:10.1007/s00401-026-03033-2)

Dunot et al.
Supplementary File

Full Blots

Figure 1b: Hippocampus AETA women Ctl vs AD (ponceau)

Gel 1

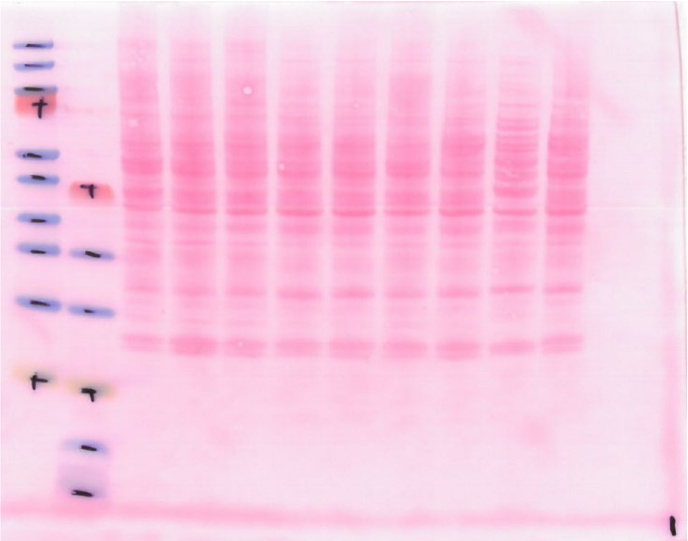

Gel 2

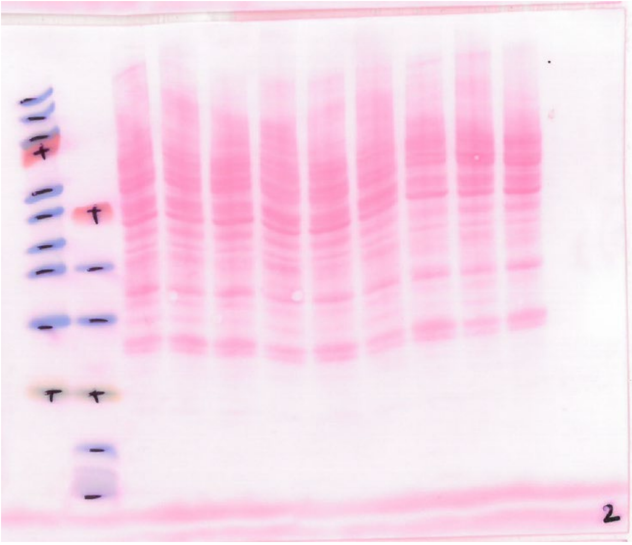

Gel 3

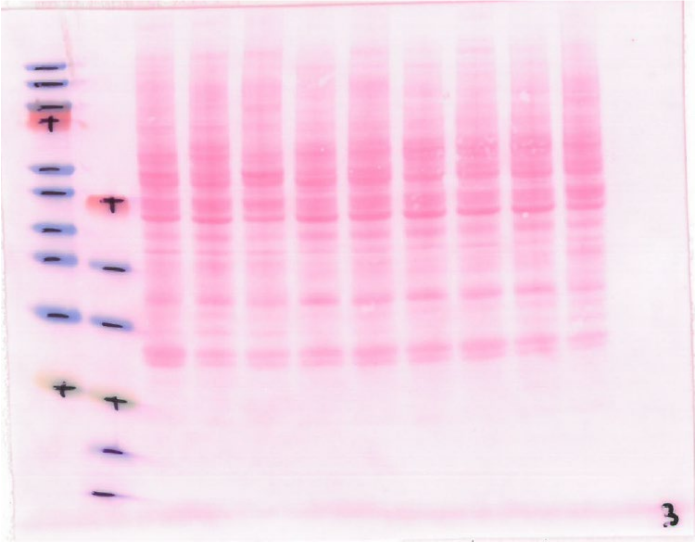

Gel 4

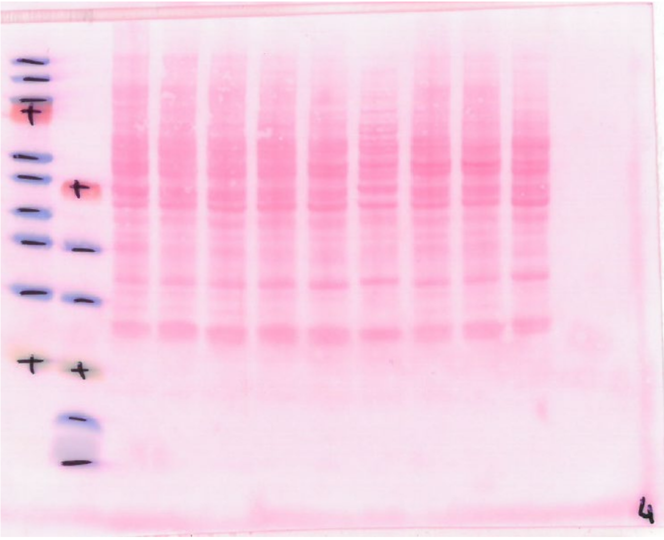

Gel 5

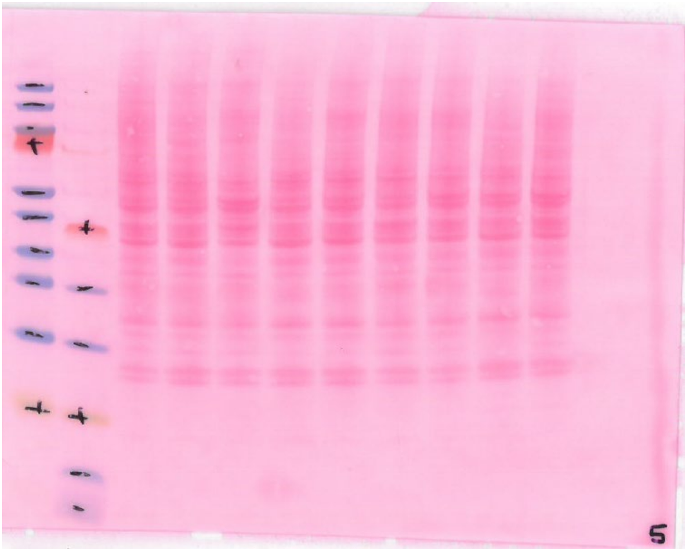

Gel 6

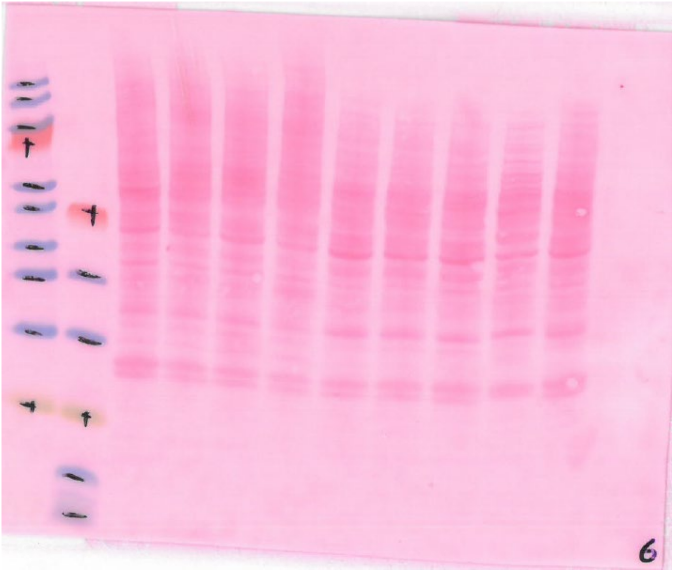

Figure 1b: Hippocampus AETA women Ctl vs AD

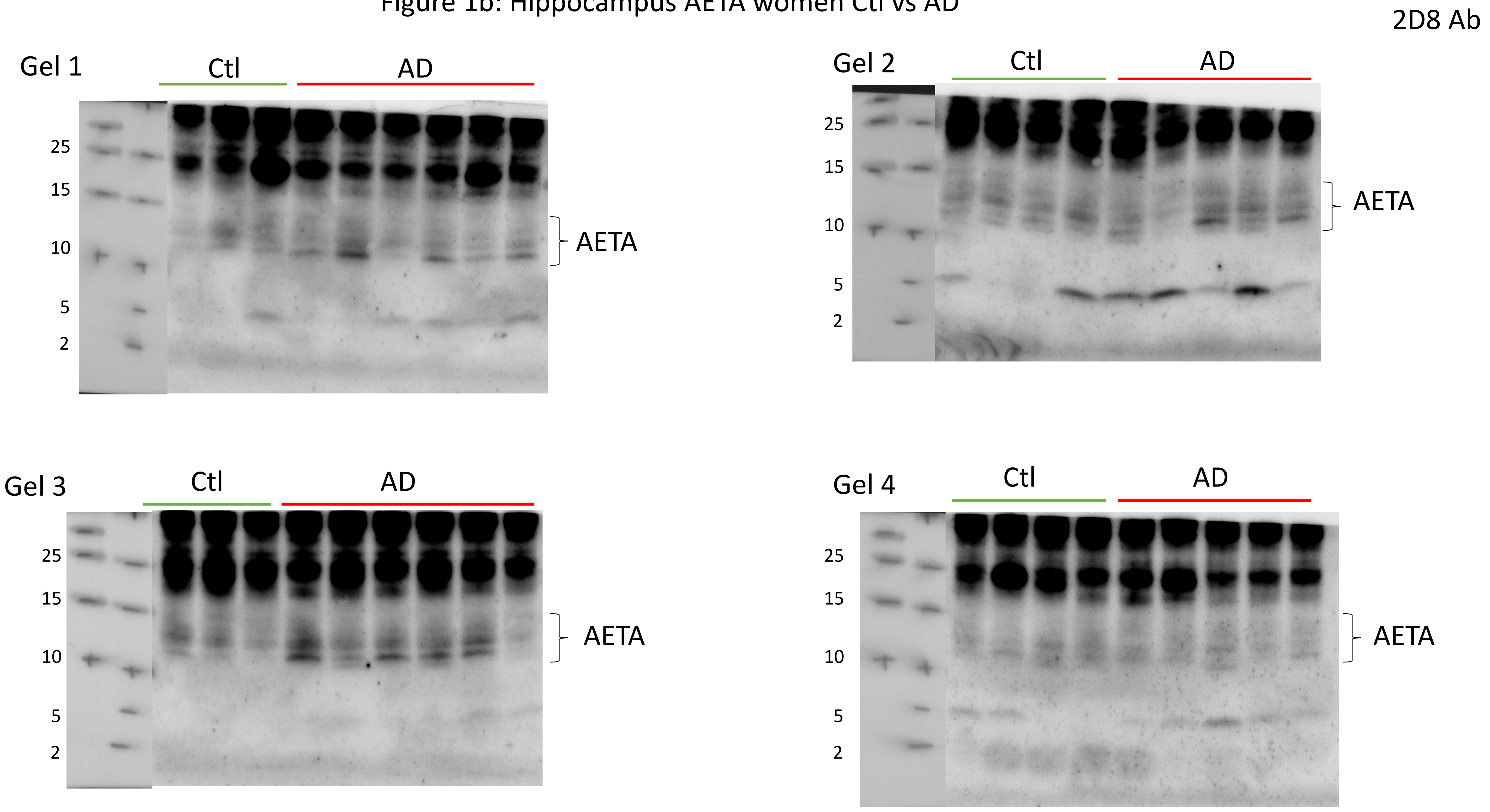

Figure 1b: Hippocampus AETA women Ctl vs AD

2D8 Ab

Gel 5

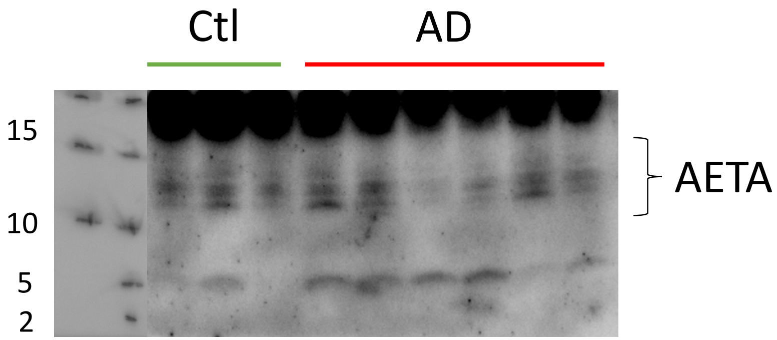

Gel 6

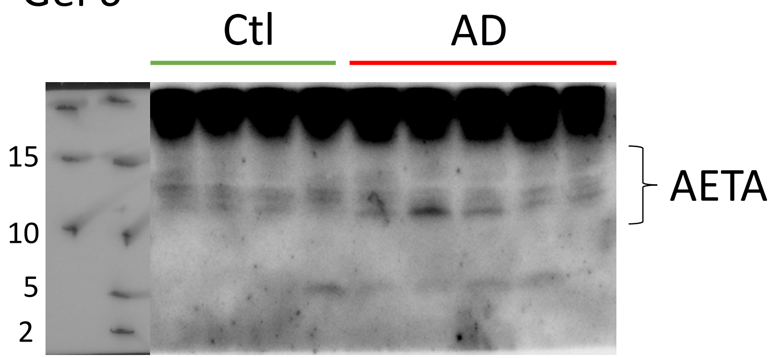

Figure 1b: Hippocampus AETA Men Ctl vs AD (ponceau)

Gel 7

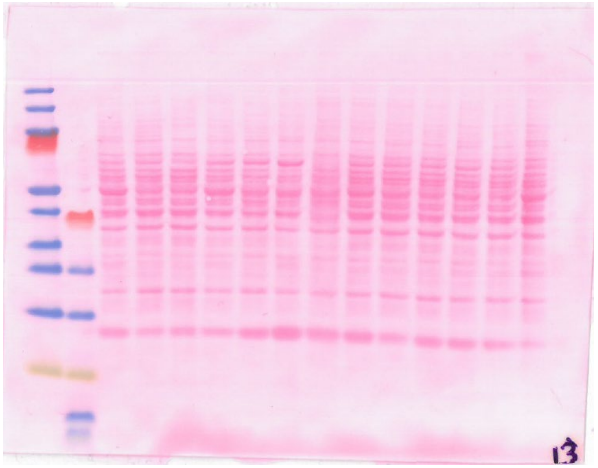

Gel 8

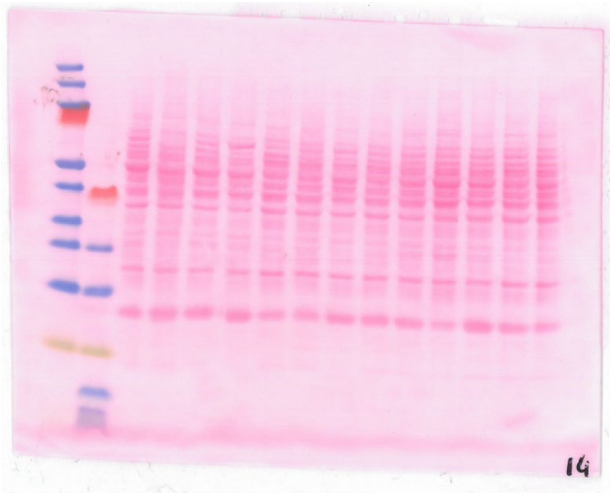

Gel 9

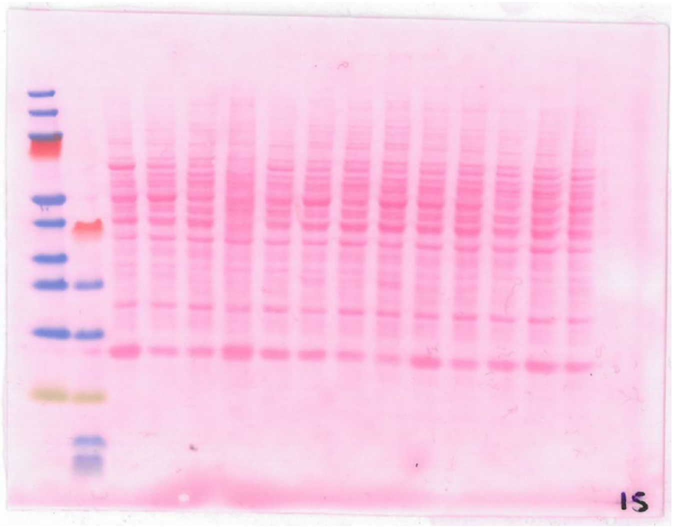

Figure 1b: Hippocampus AETA Men Ctl vs AD

2D8 Ab

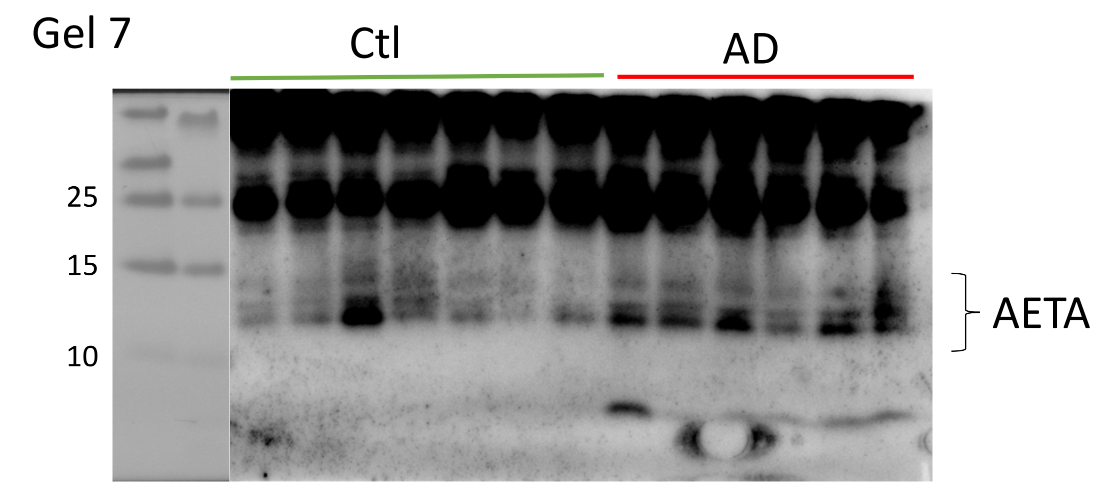

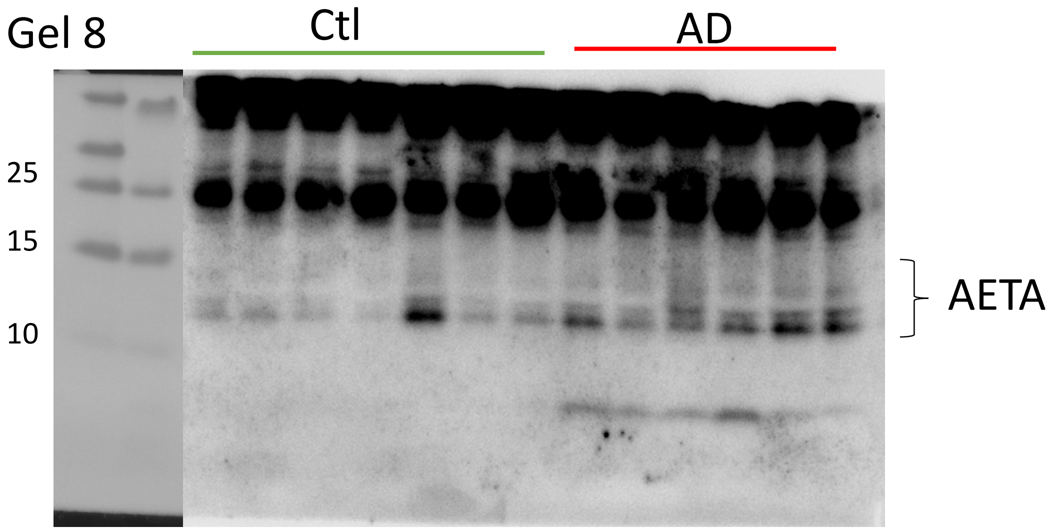

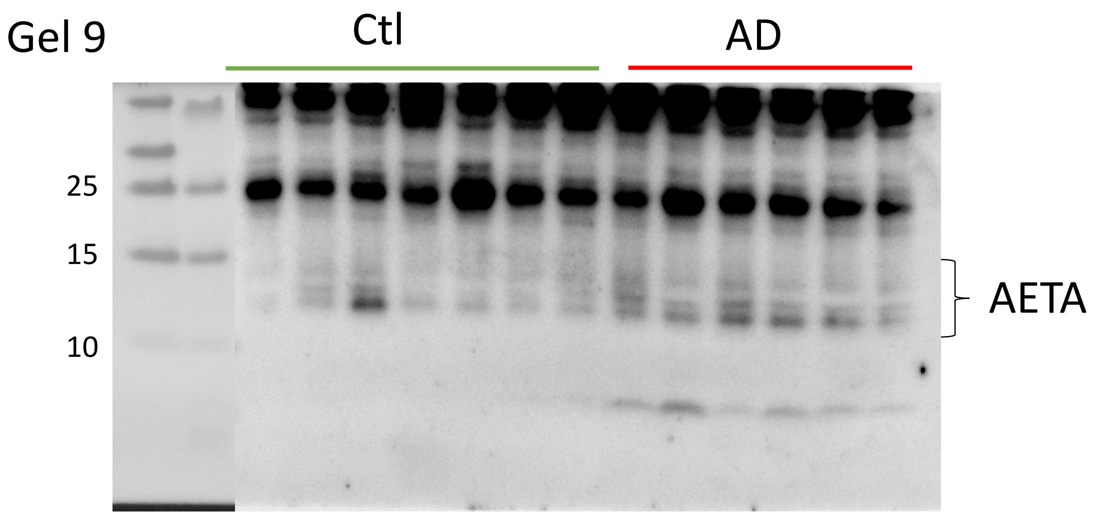

Figure S1b : PFC AETA Women Ctl vs AD

2E9 Ab

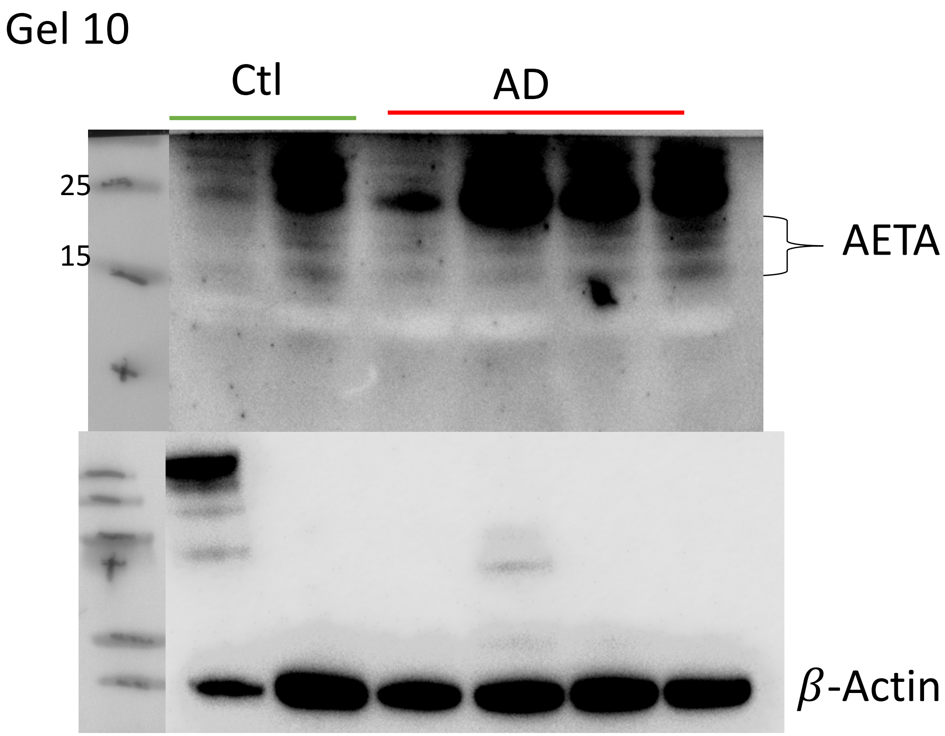

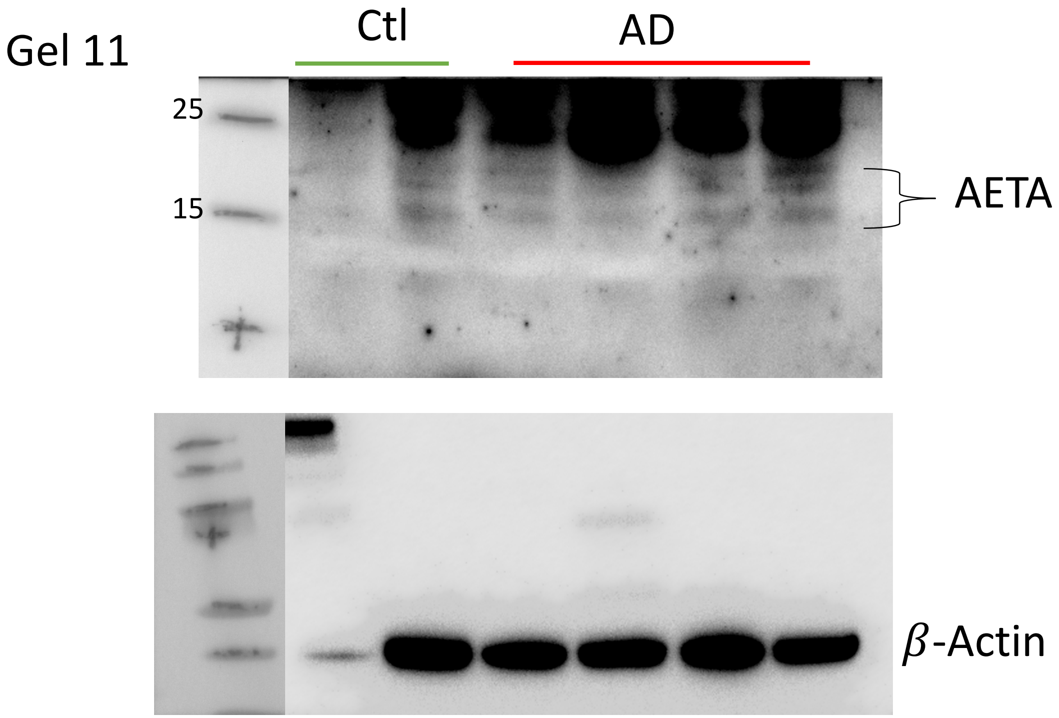

Figure S1b : PFC AETA Women Ctl vs AD

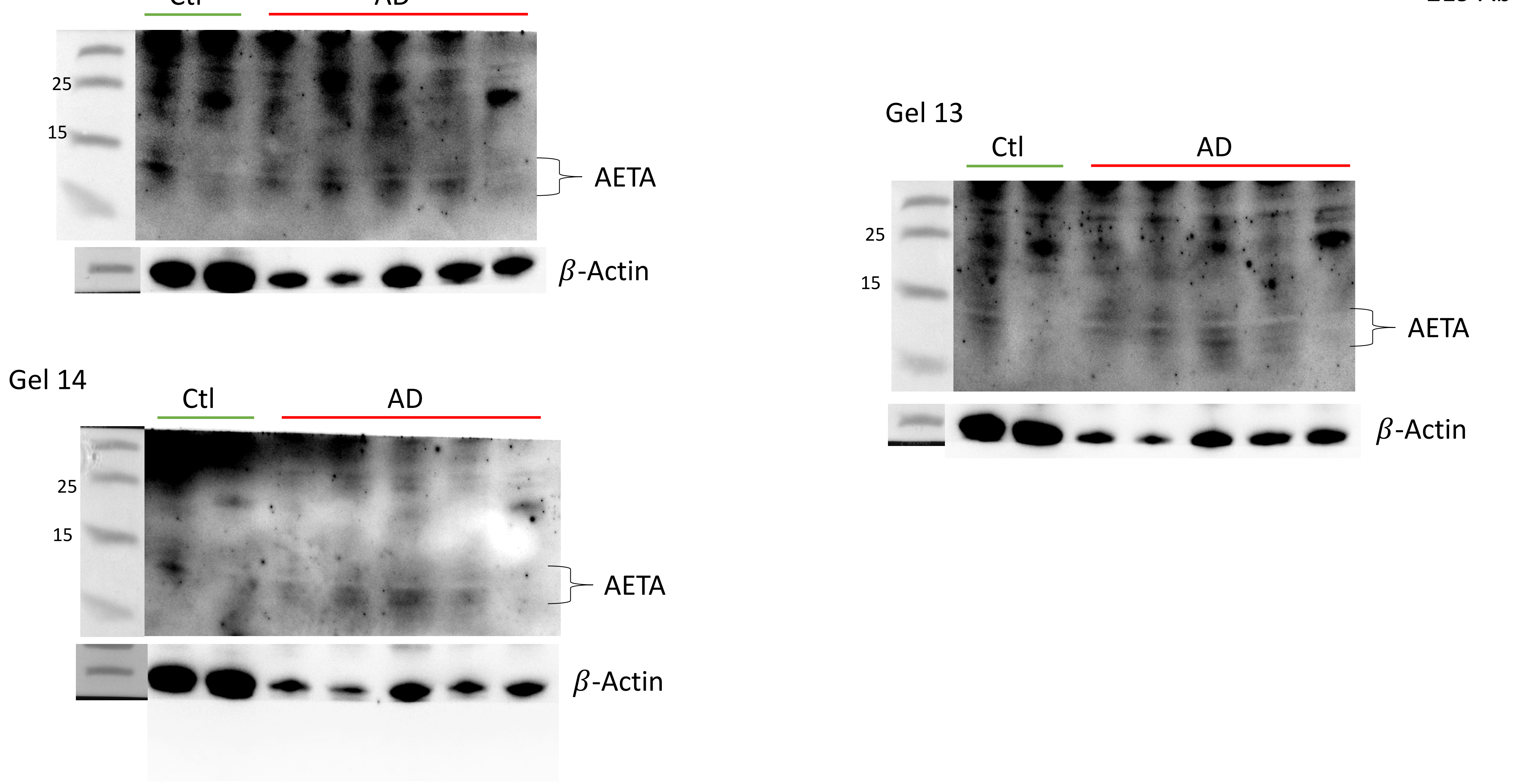

Figure S1b : PFC AETA men Ctl vs AD

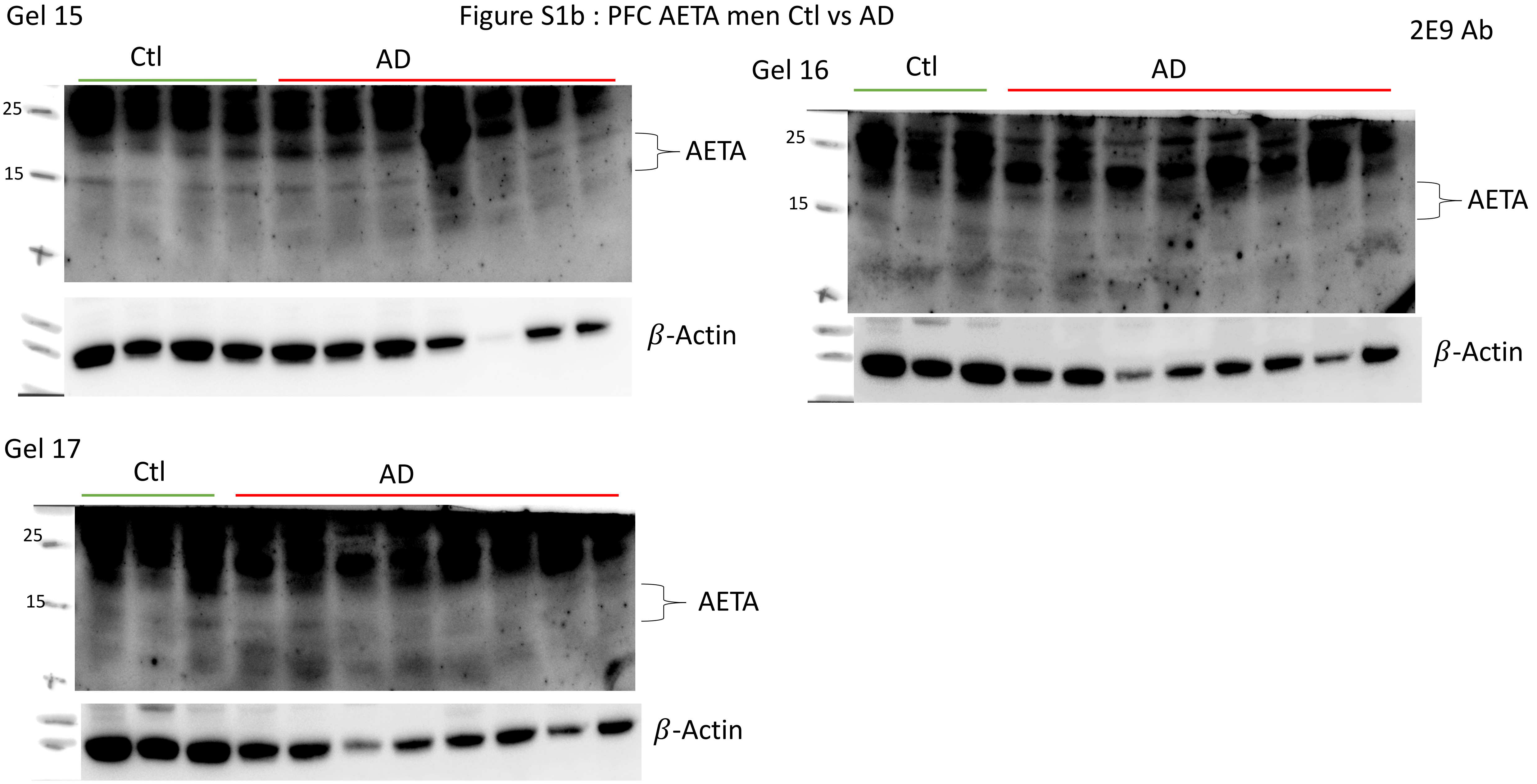

Figure 1c: Hippocampus APP women Ctl vs AD (ponceau)

Gel 18

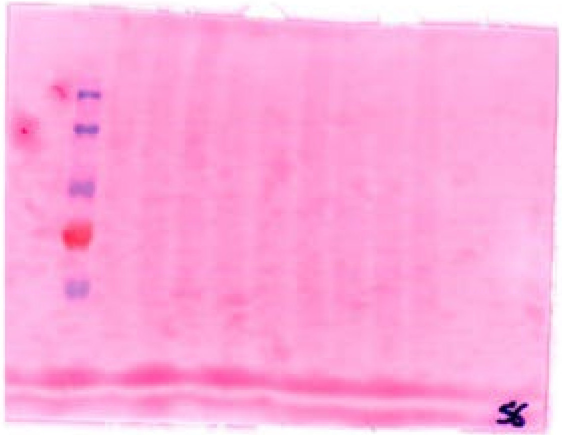

Gel 19

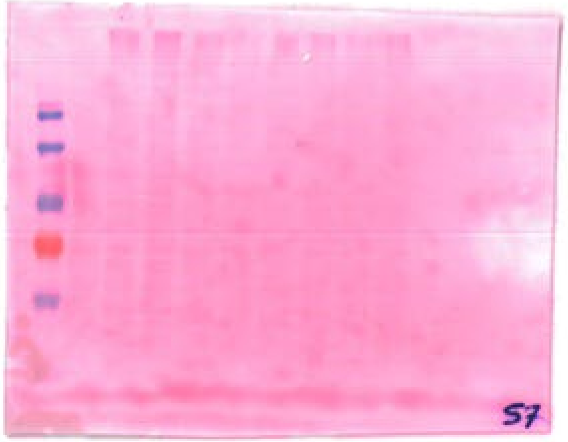

Gel 20

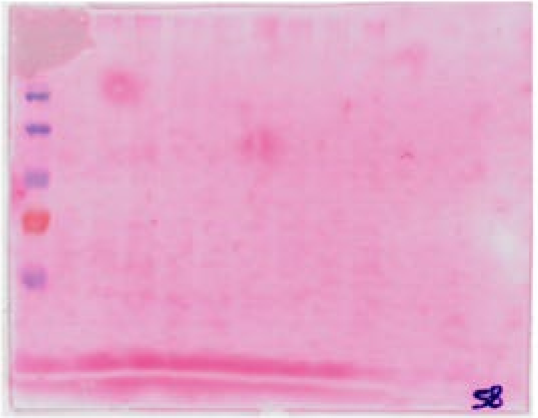

Gel 21

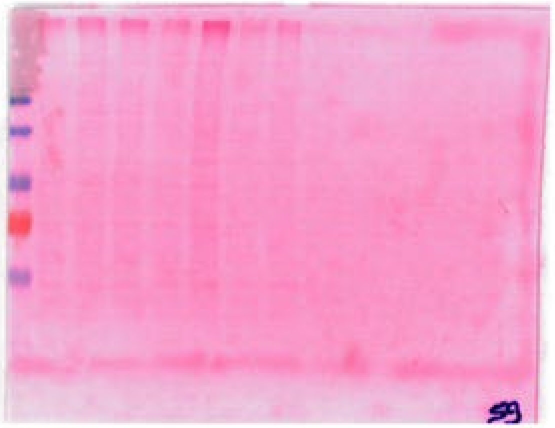

Gel 22

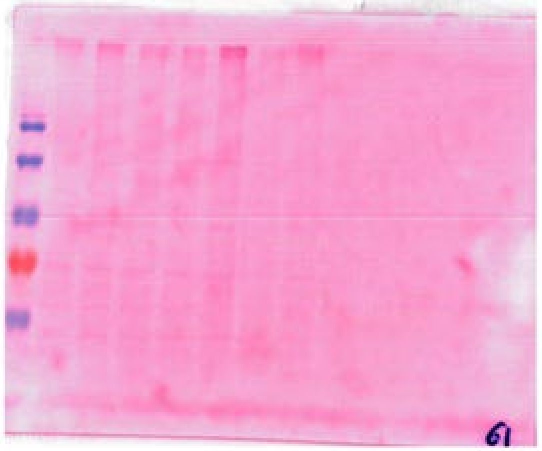

Figure 1c: Hippocampus APP women Ctl vs AD

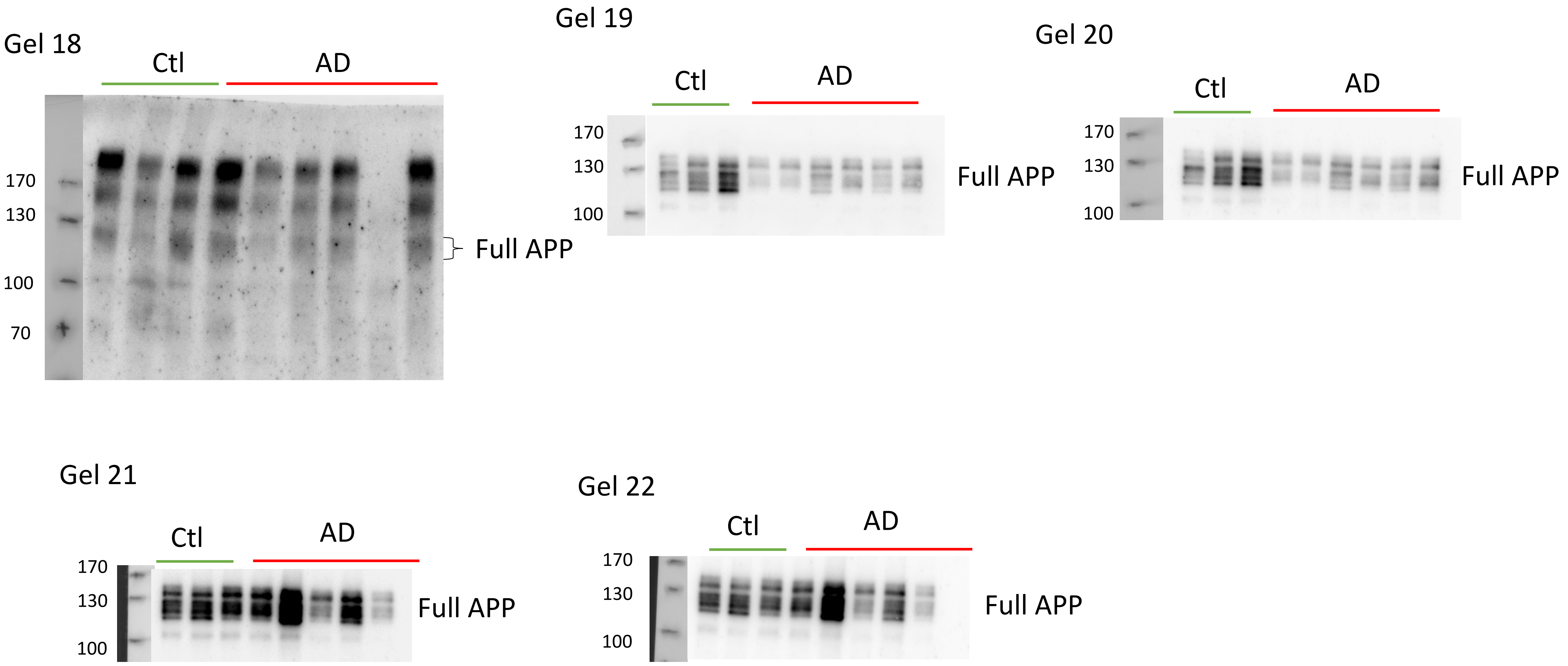

Figure 1c: Hippocampus APP men Ctl vs AD (ponceau)

Gel 23

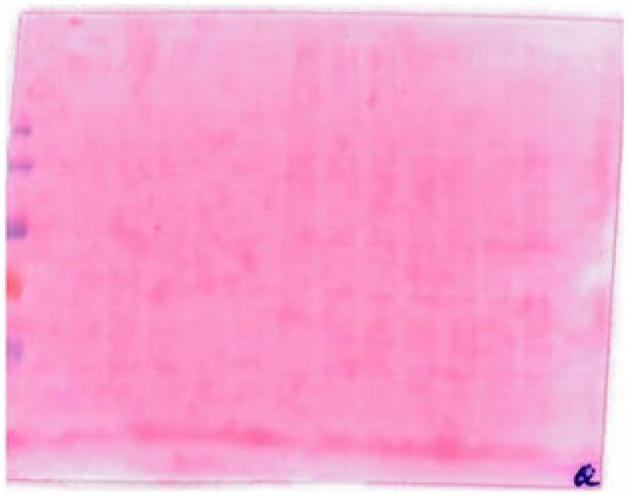

Gel 24

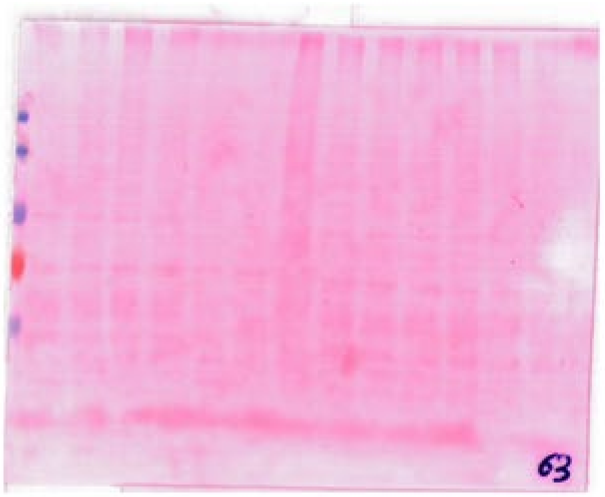

Gel 25

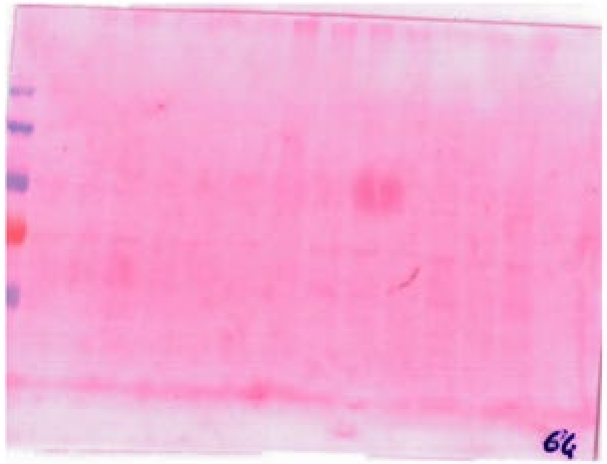

Figure 1c: Hippocampus APP men Ctl vs AD

Gel 23

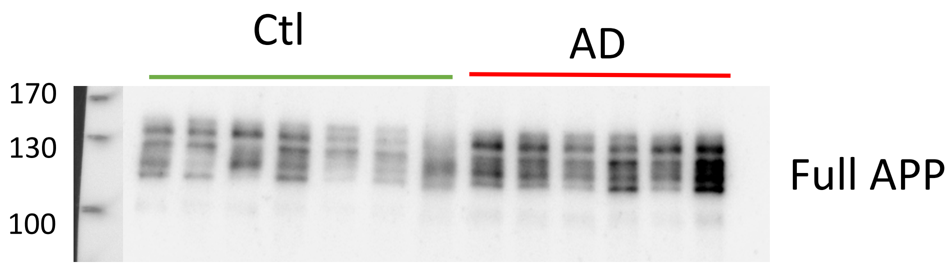

Gel 24

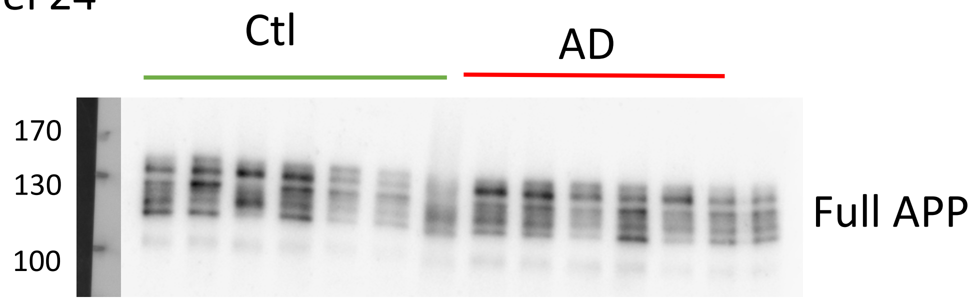

Gel 25

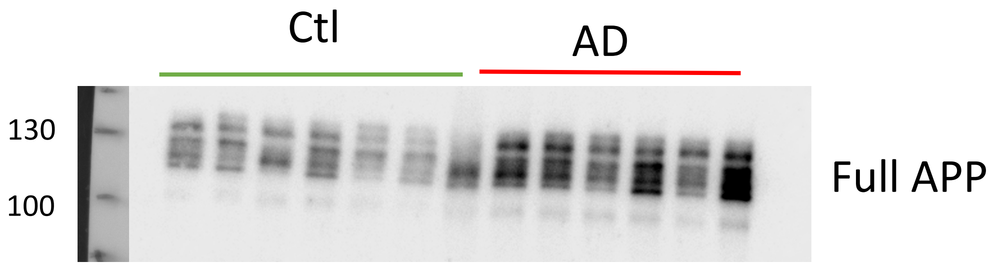

Figure 1d: Hippocampus AETA men vs women AD (Ponceau)

Gel 29

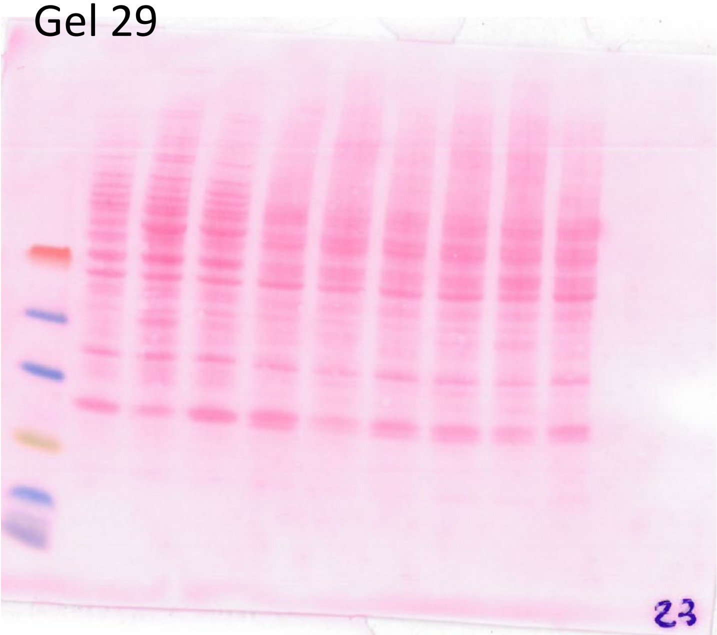

Gel 30

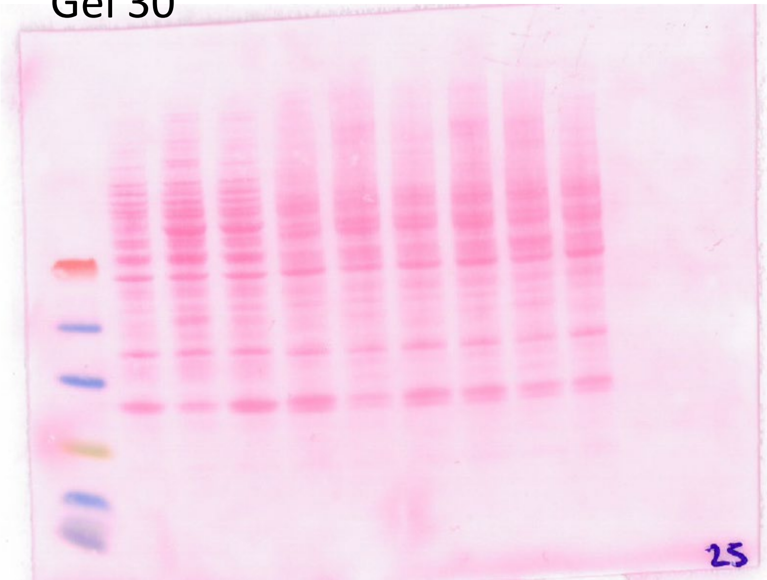

Gel 31

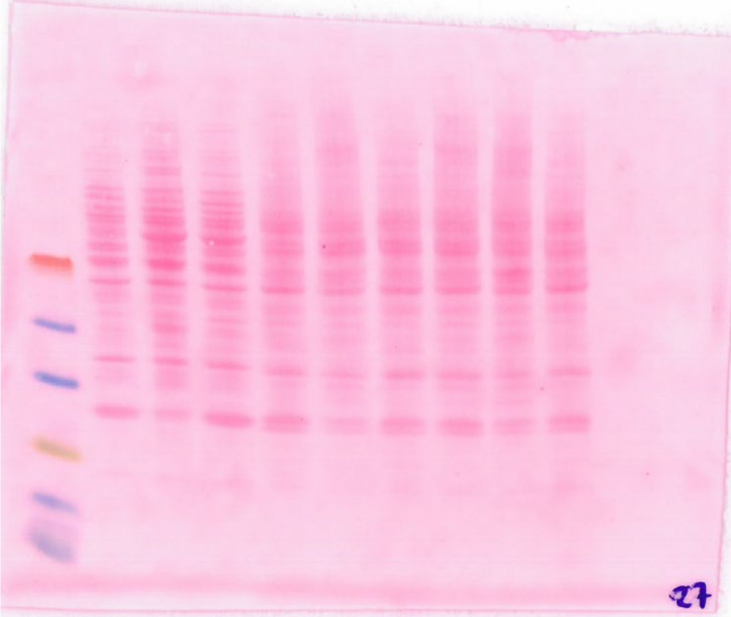

Gel 32

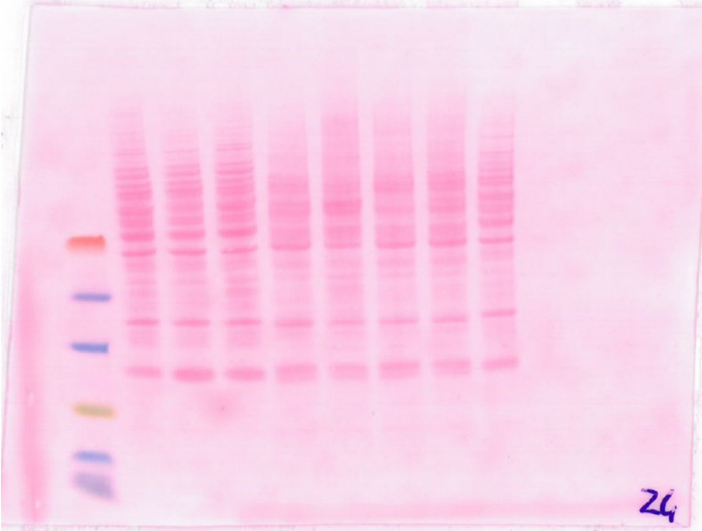

Gel 33

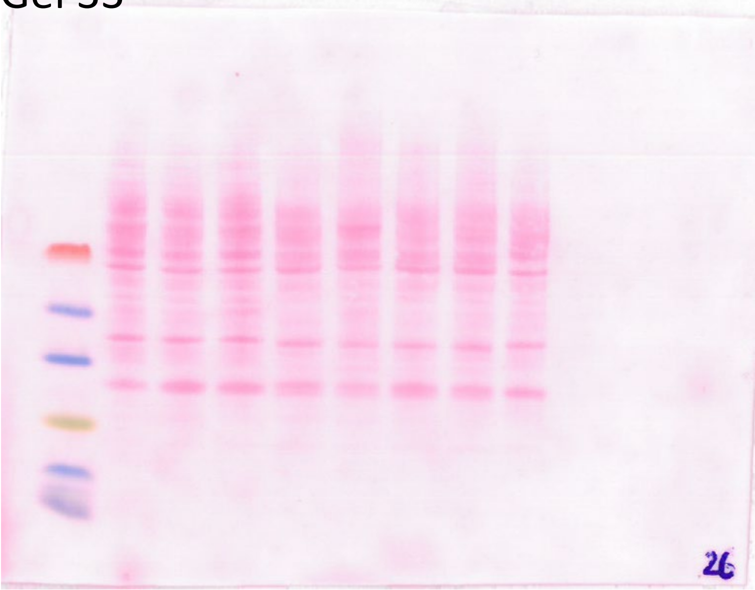

Gel 34

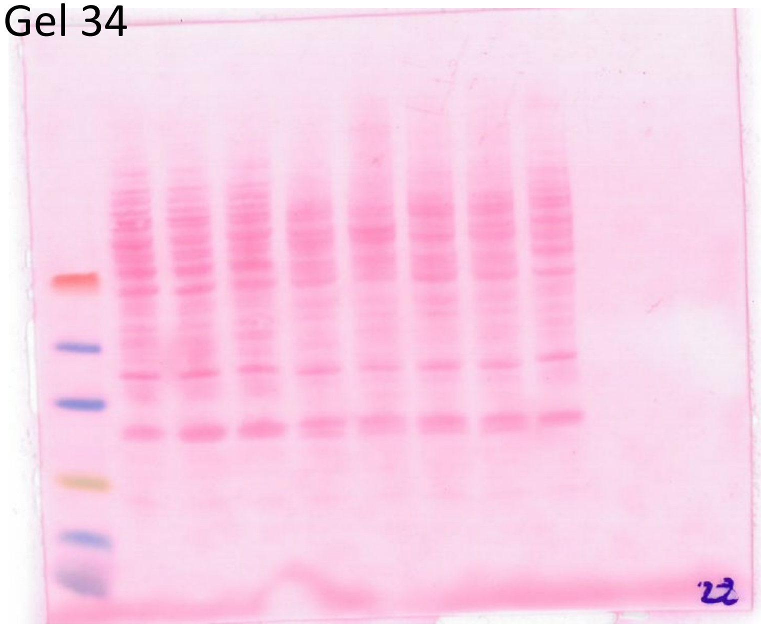

Figure 1d: Hippocampus AETA men vs women AD

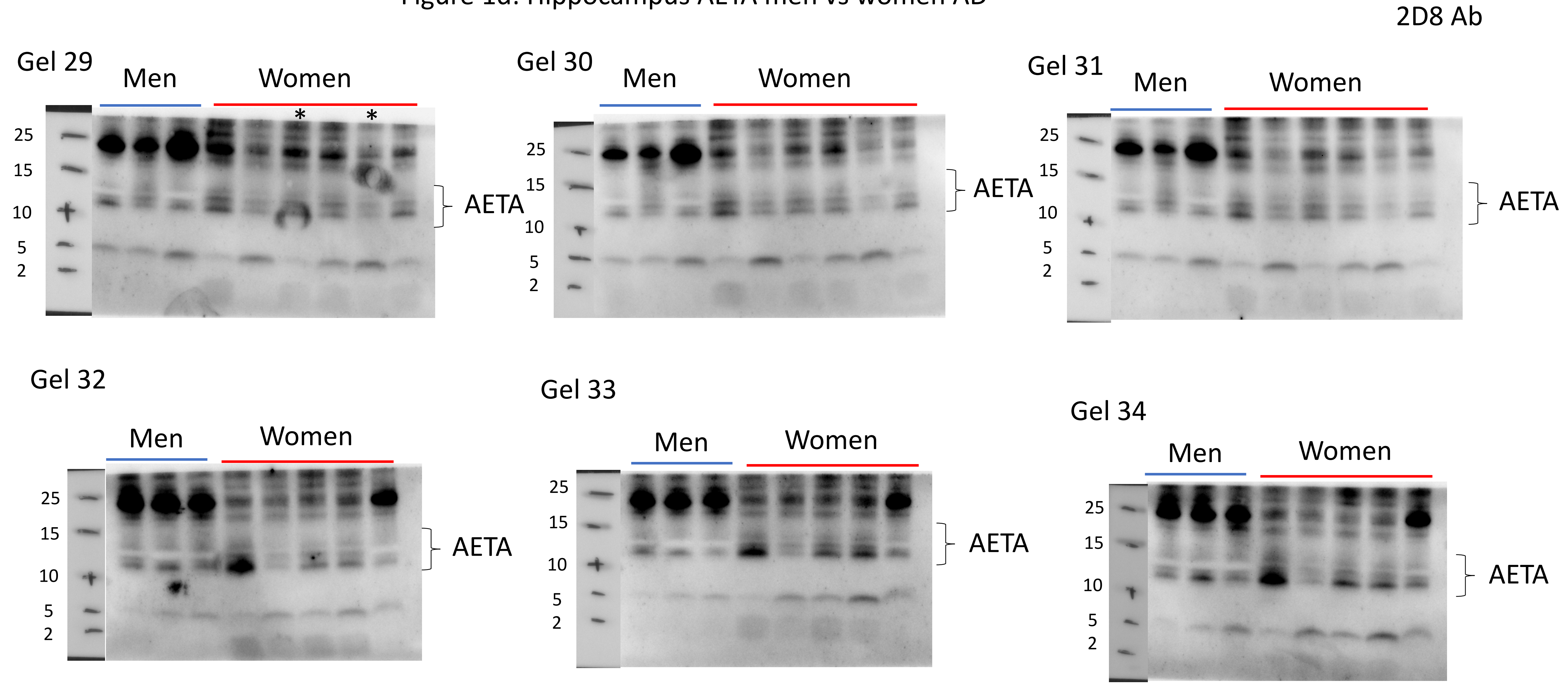

Figure S1e: Hippocampus AETA men vs women ctl (Ponceau)

Gel 26

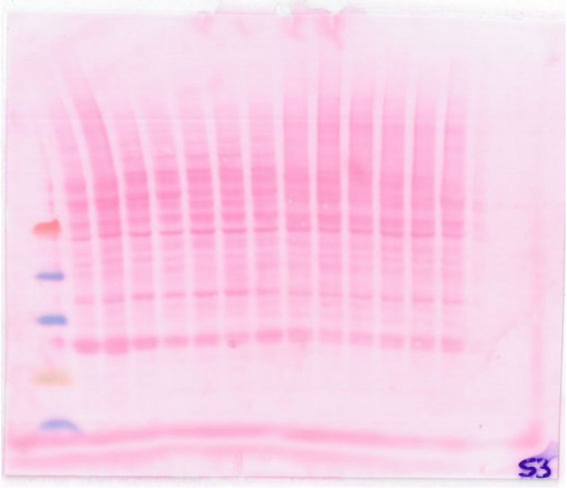

Gel 27

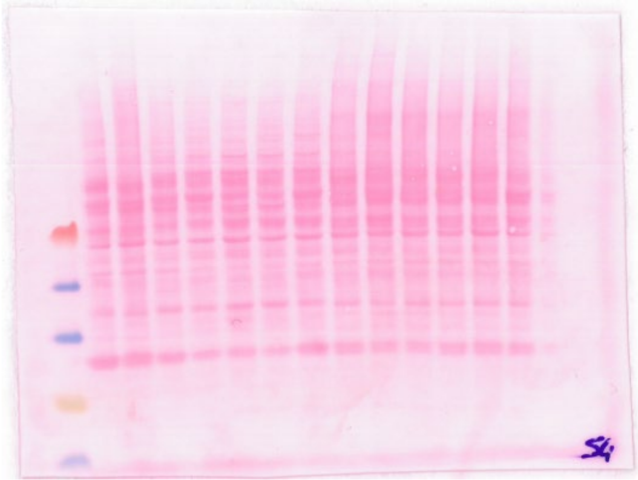

Gel 28

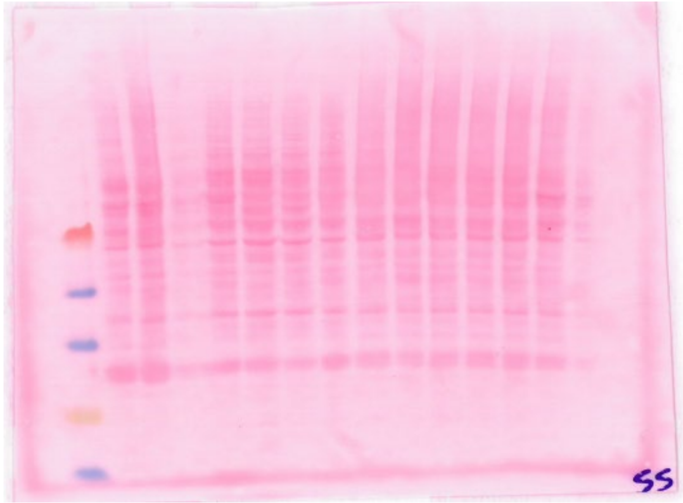

Figure S1e: Hippocampus AETA men vs women ctl

2D8 Ab

Gel 26

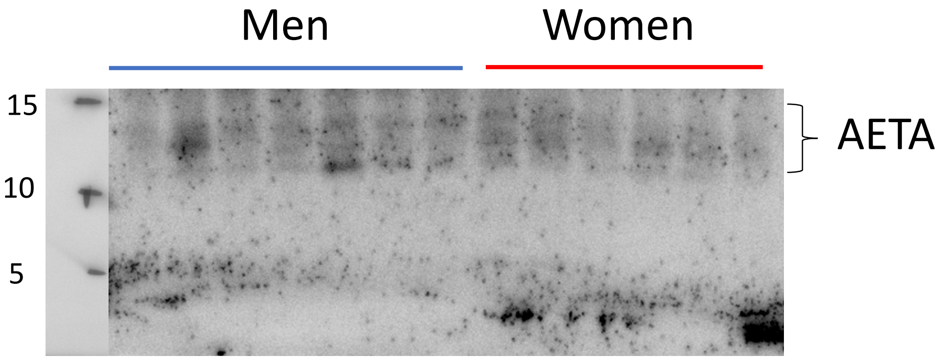

Gel 27

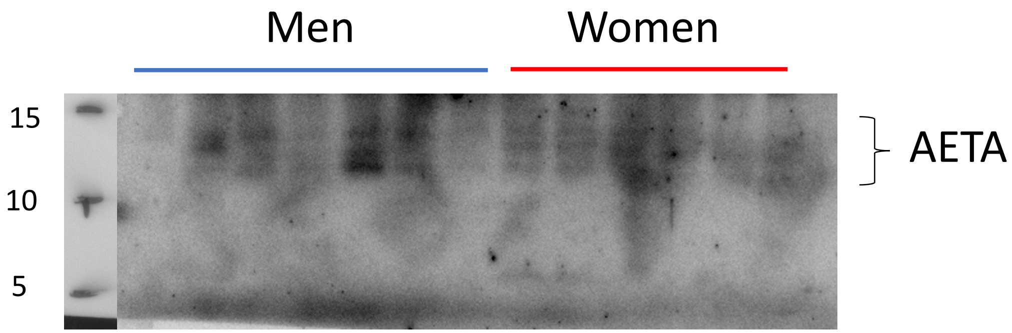

Gel 28

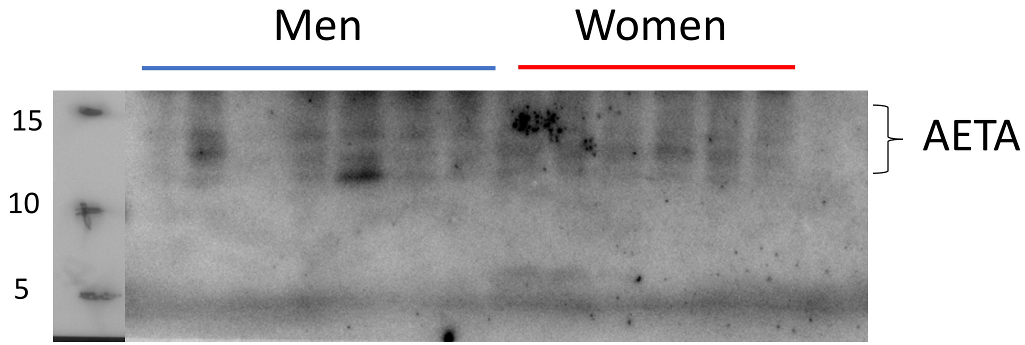

Figure S1a: Hippocampus GAPDH women ctl vs AD

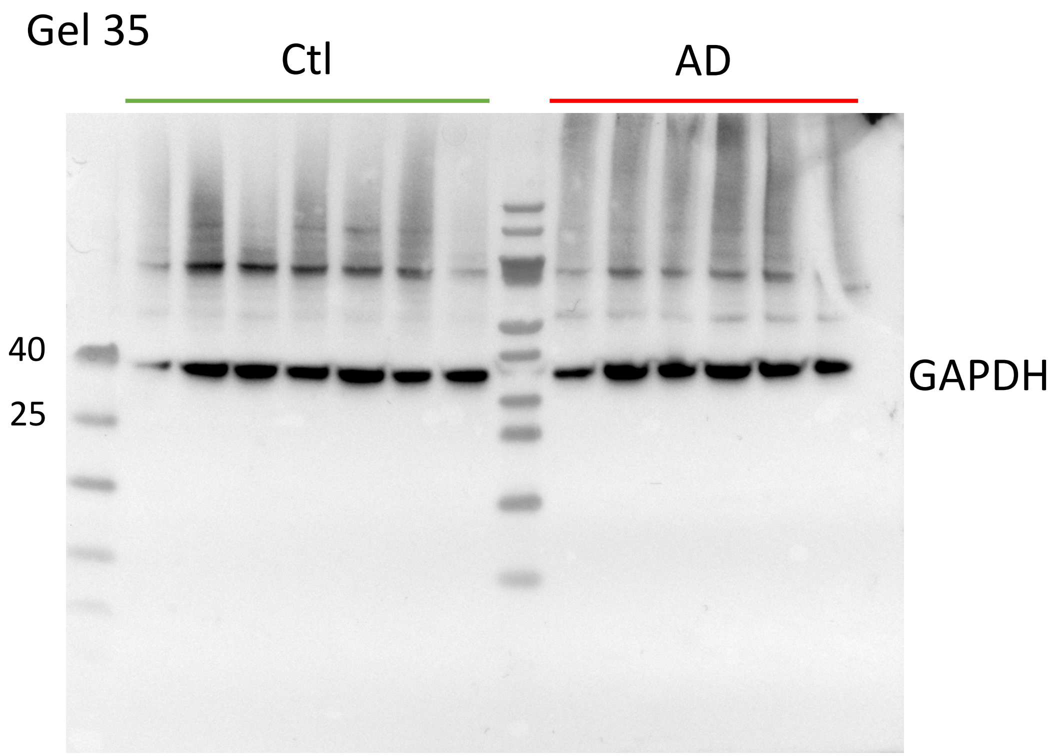

Figure 2b: human AETA at different ages in hippocampi of AETA-m mice

2E9 Ab

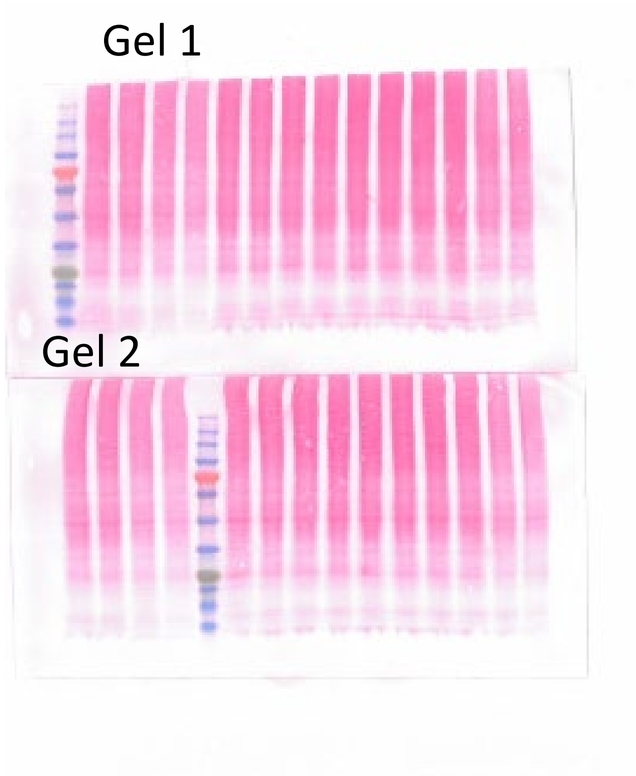

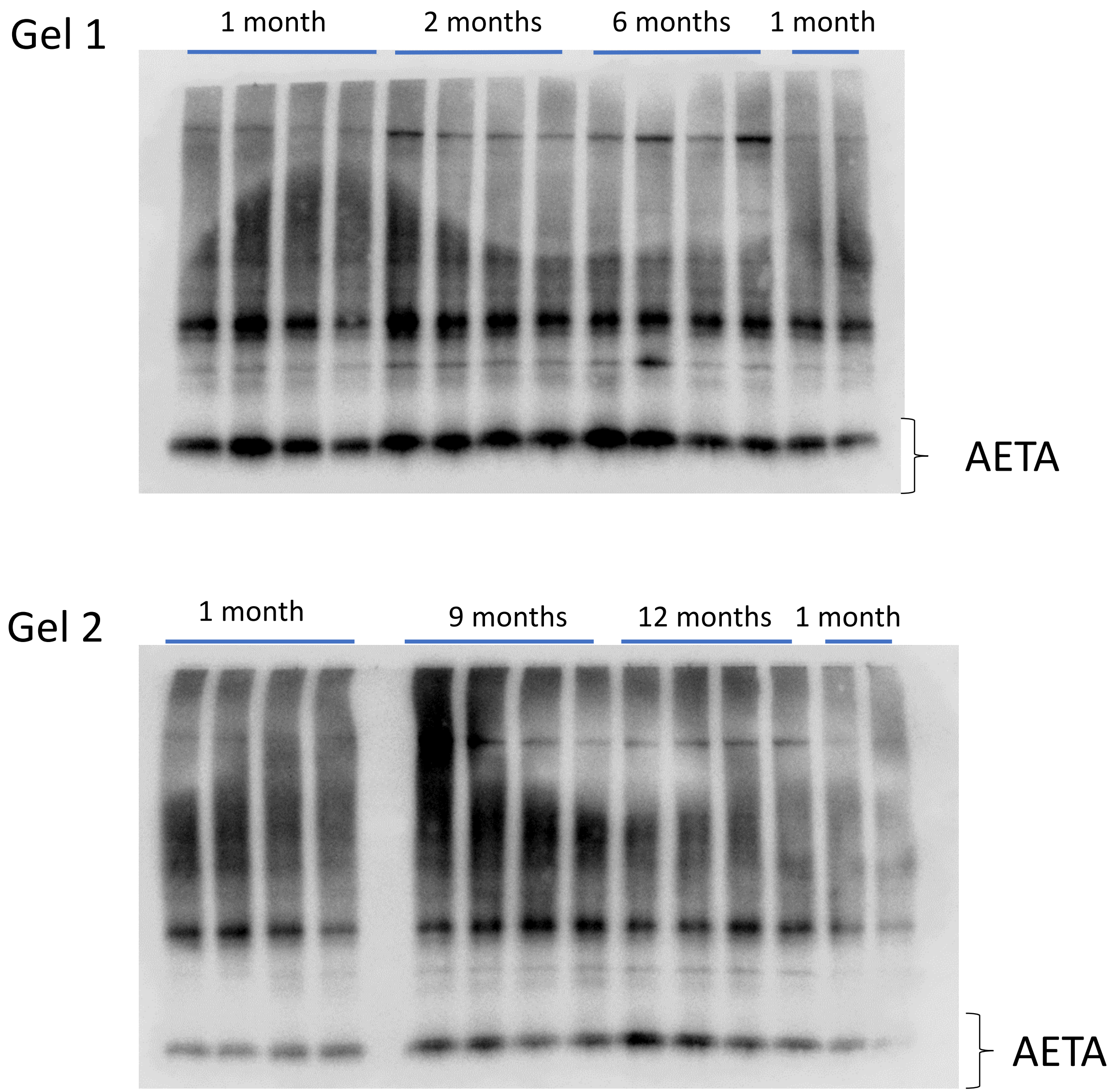

Figure S2e: human AETA in hippocampi of AETA-m mice of males and females (ponceau)

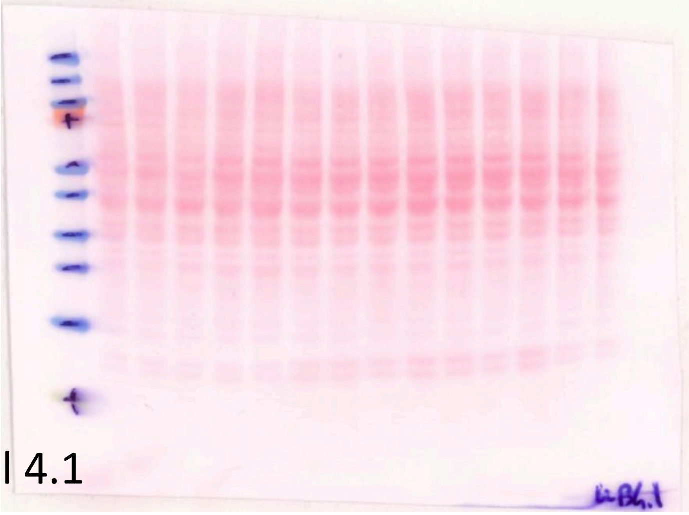
Gel 4.1

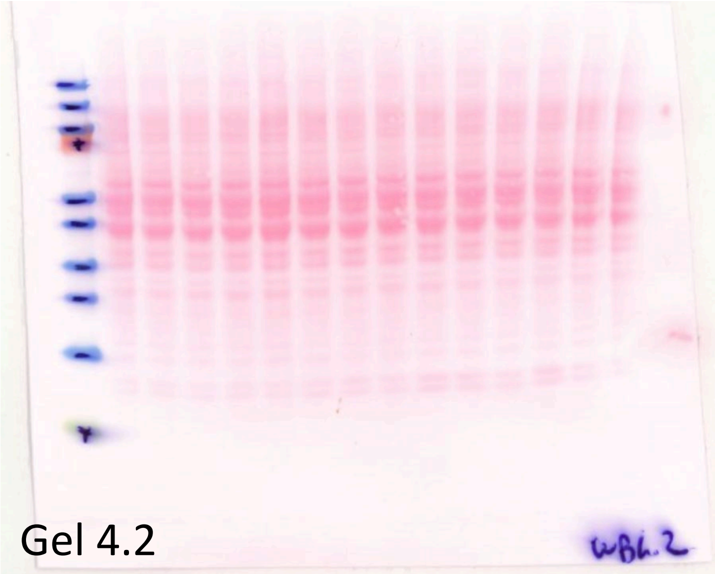
Gel 4.2

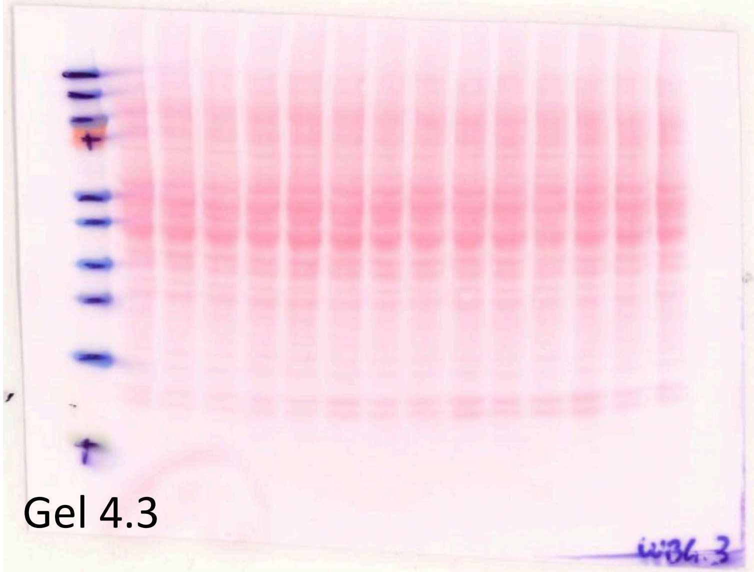
Gel 4.3

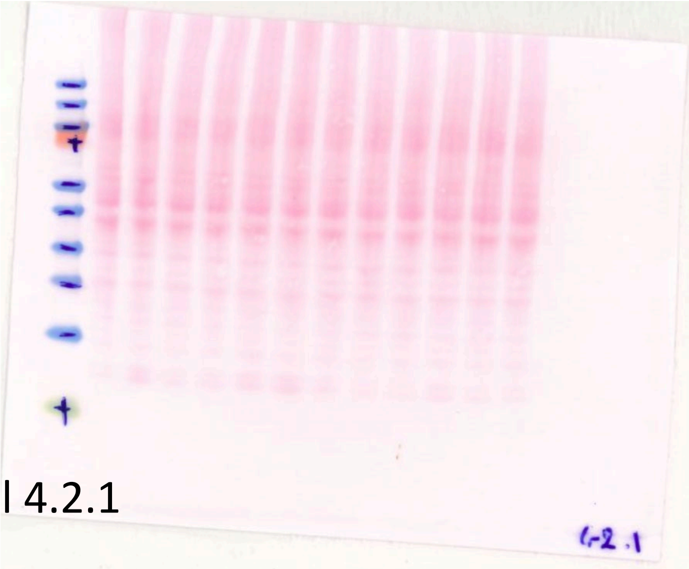
Gel 4.2.1

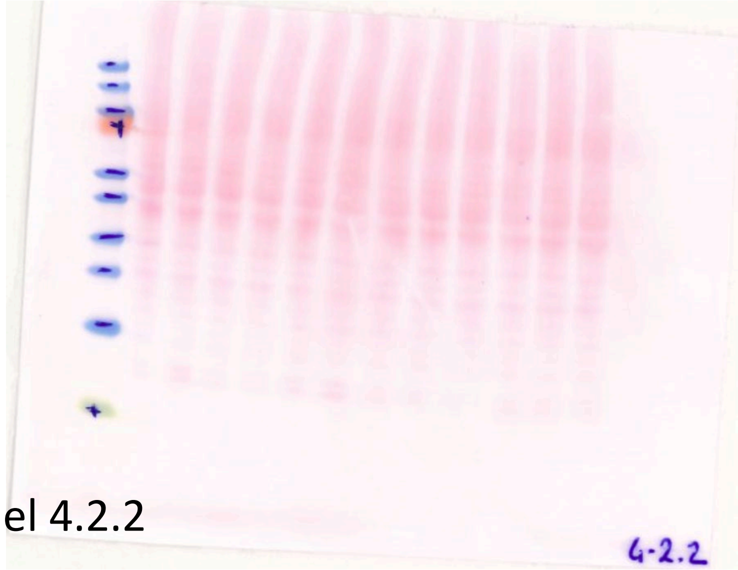
Gel 4.2.2

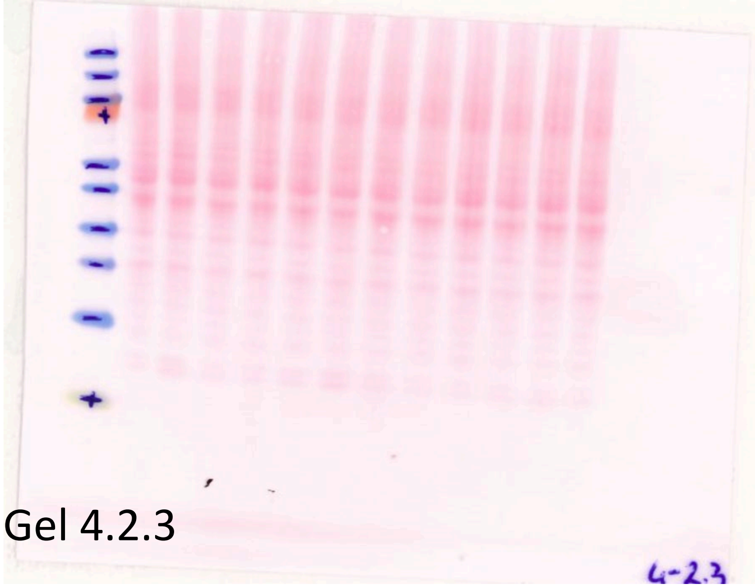
Gel 4.2.3

Figure S2e: human AETA in hippocampi of AETA-m mice of males and females

2E9 Ab

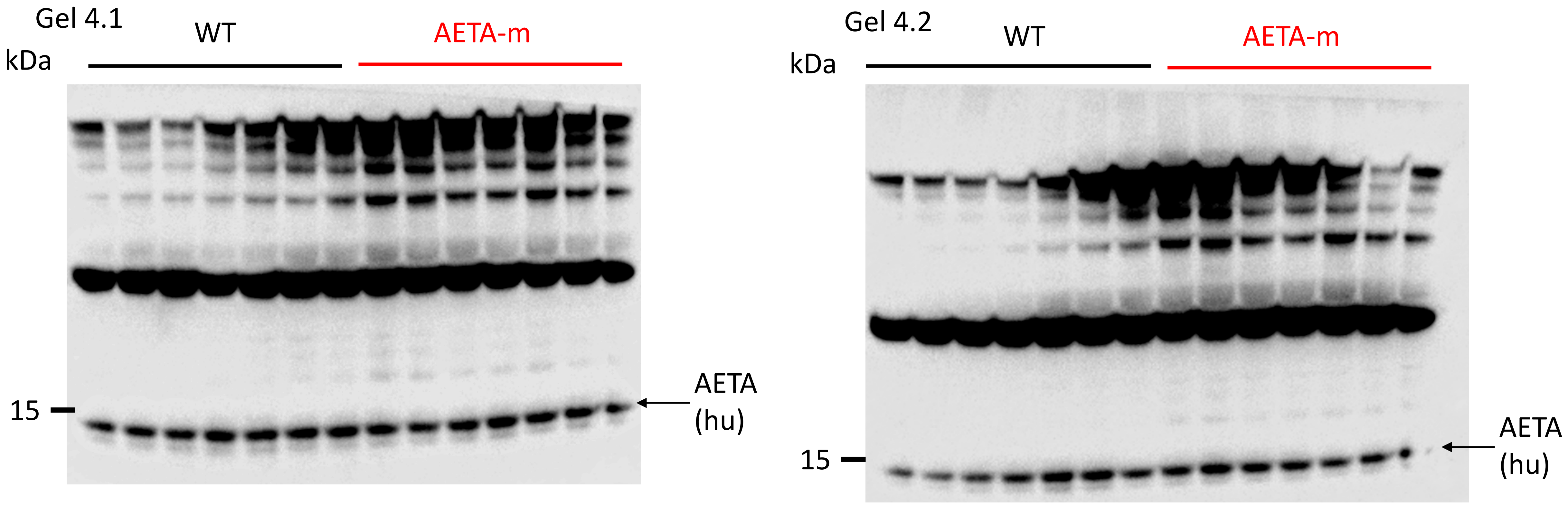

Figure S2e: human AETA in hippocampi of AETA-m mice of males and females

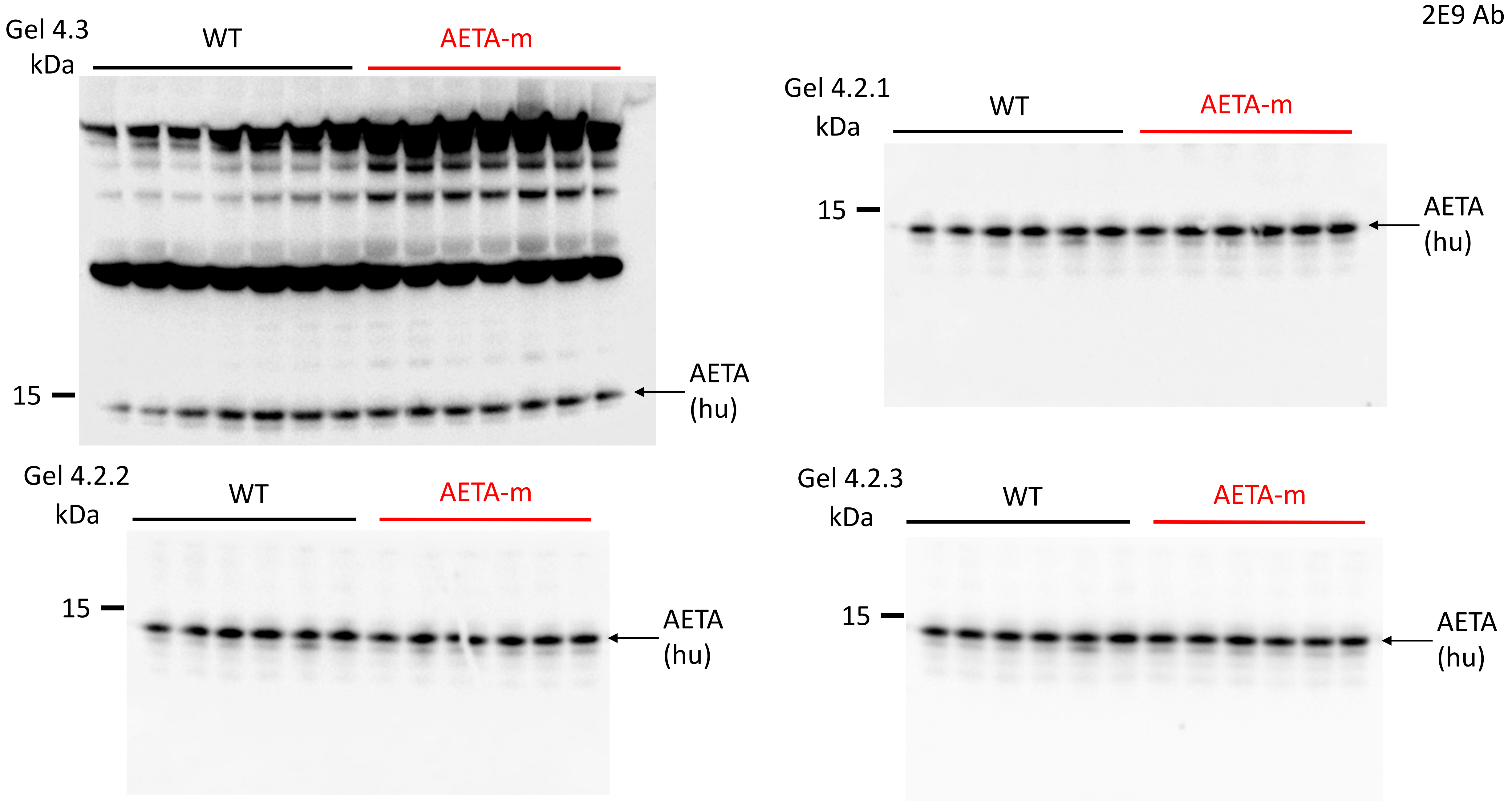

Figure S2g: endogenous CTF-η in AETA-m mice

M3.2 Ab

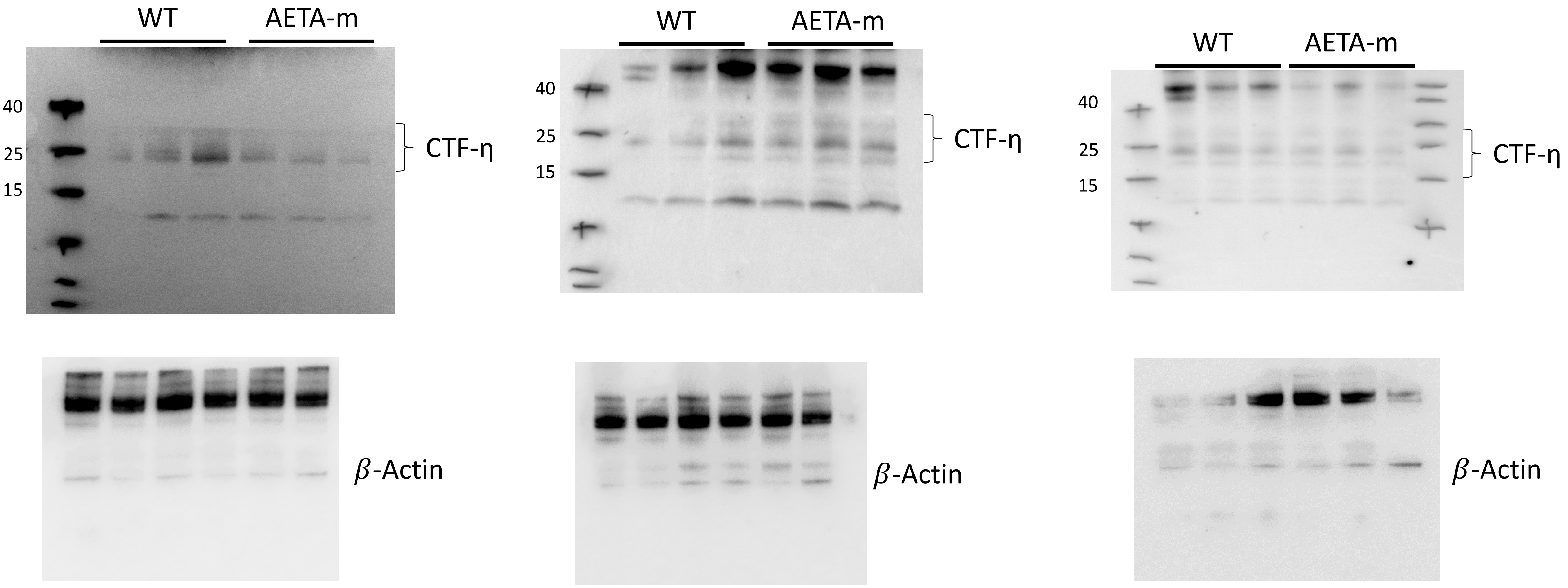

Figure S2g: APP-FL in AETA-m mice

Y188 Ab

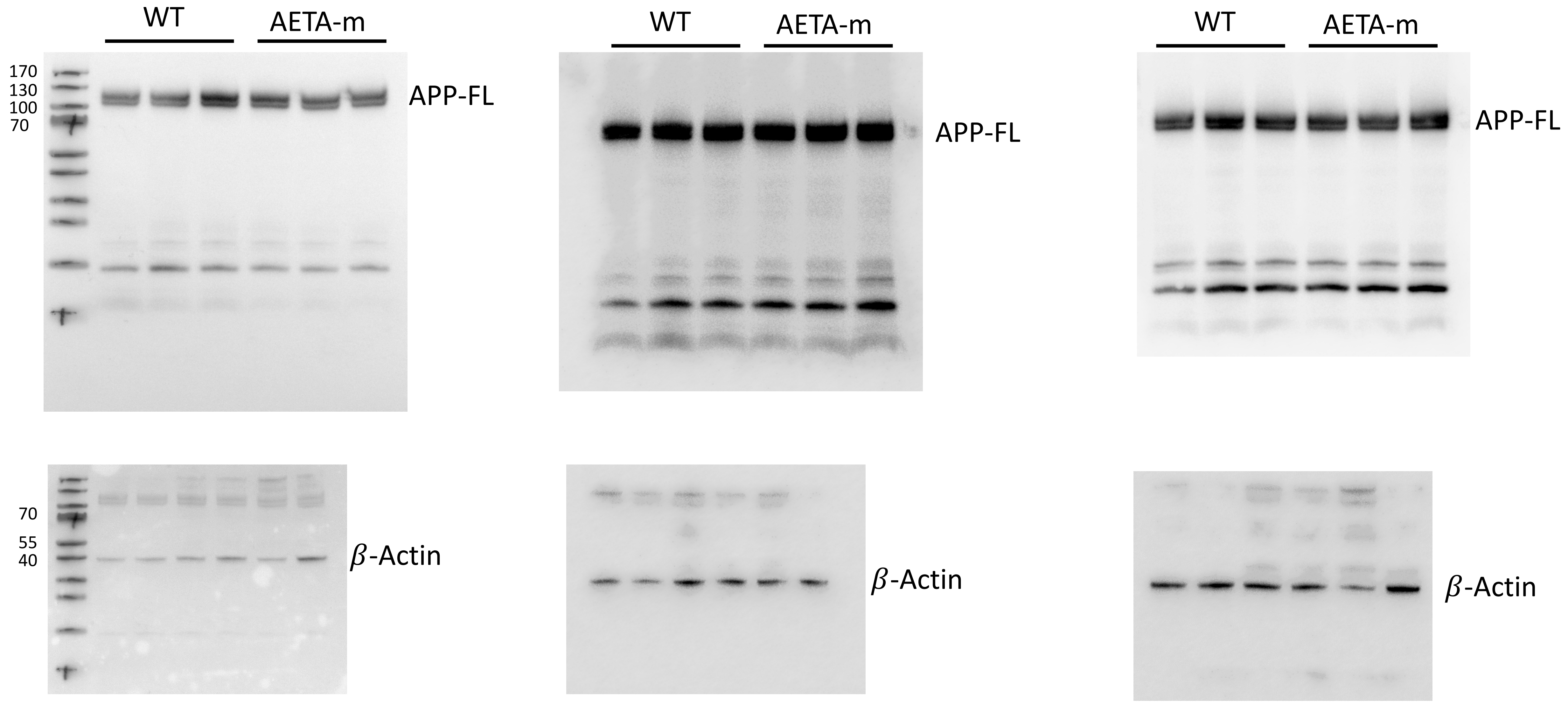

Figure S2g: sAPPα/β in AETA-m mice

22C11 Ab

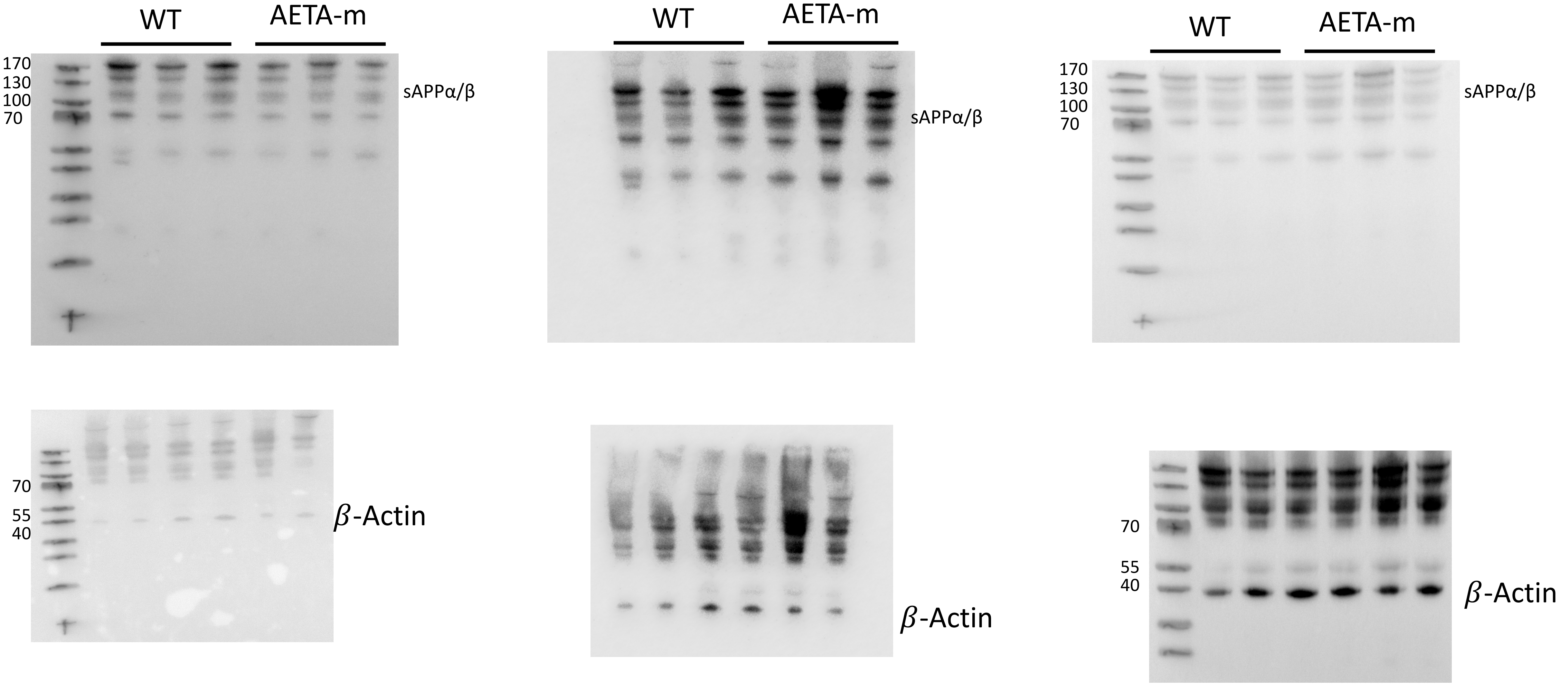

Figure S3a: C-ter Tau in AETA-m mice

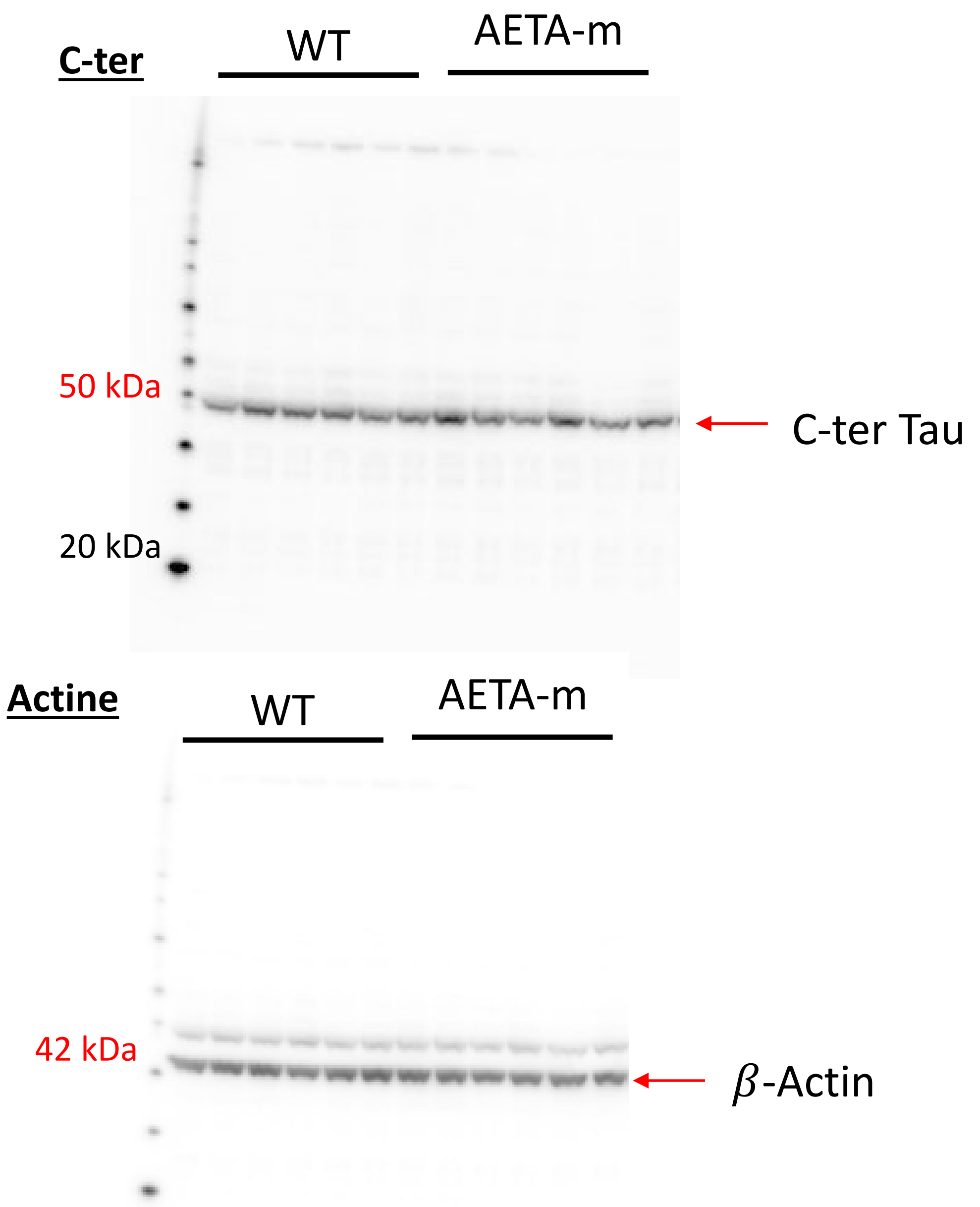

Figure S3b: Tau-1 in AETA-m mice

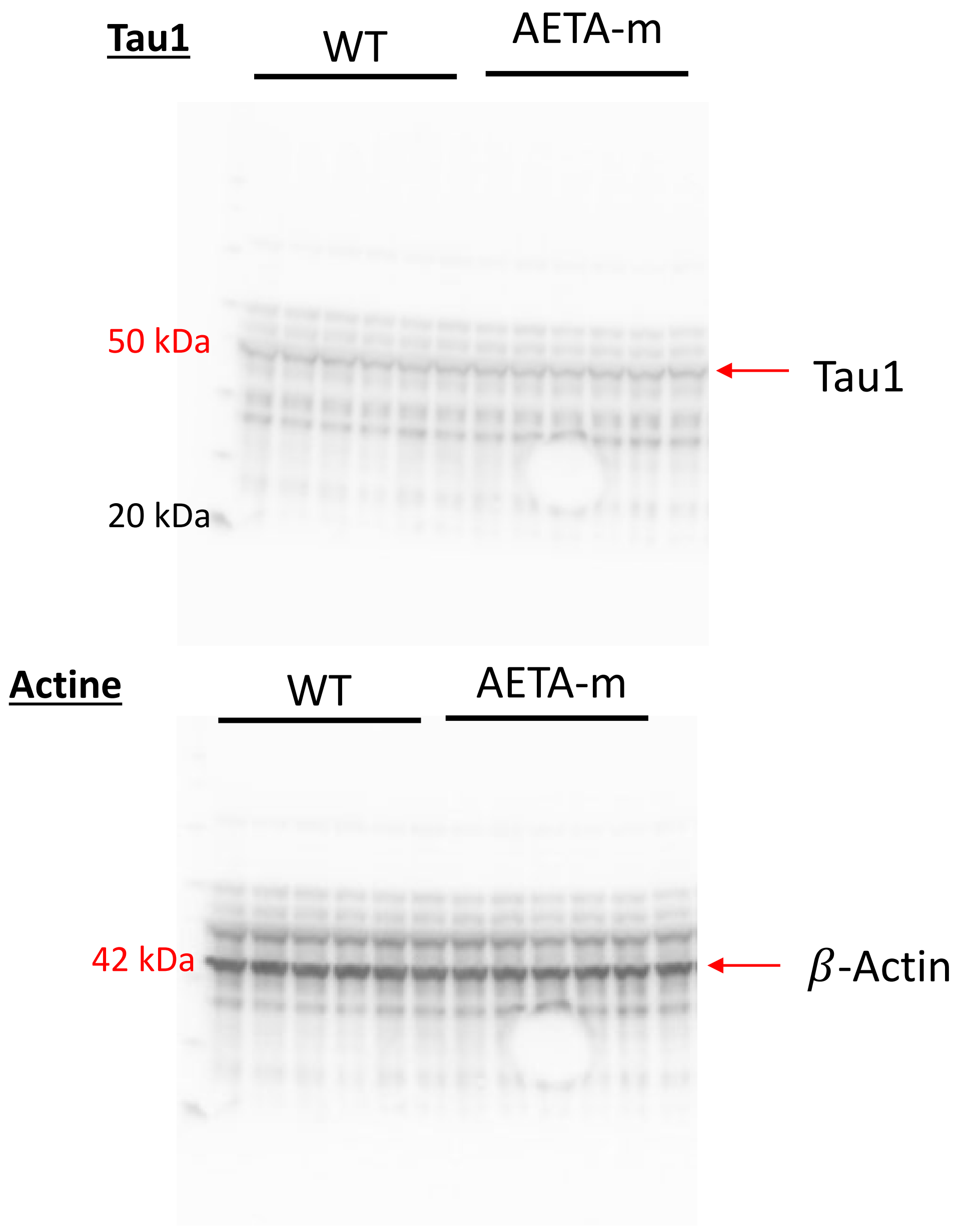

Figure S3c: pS199 in AETA-m mice

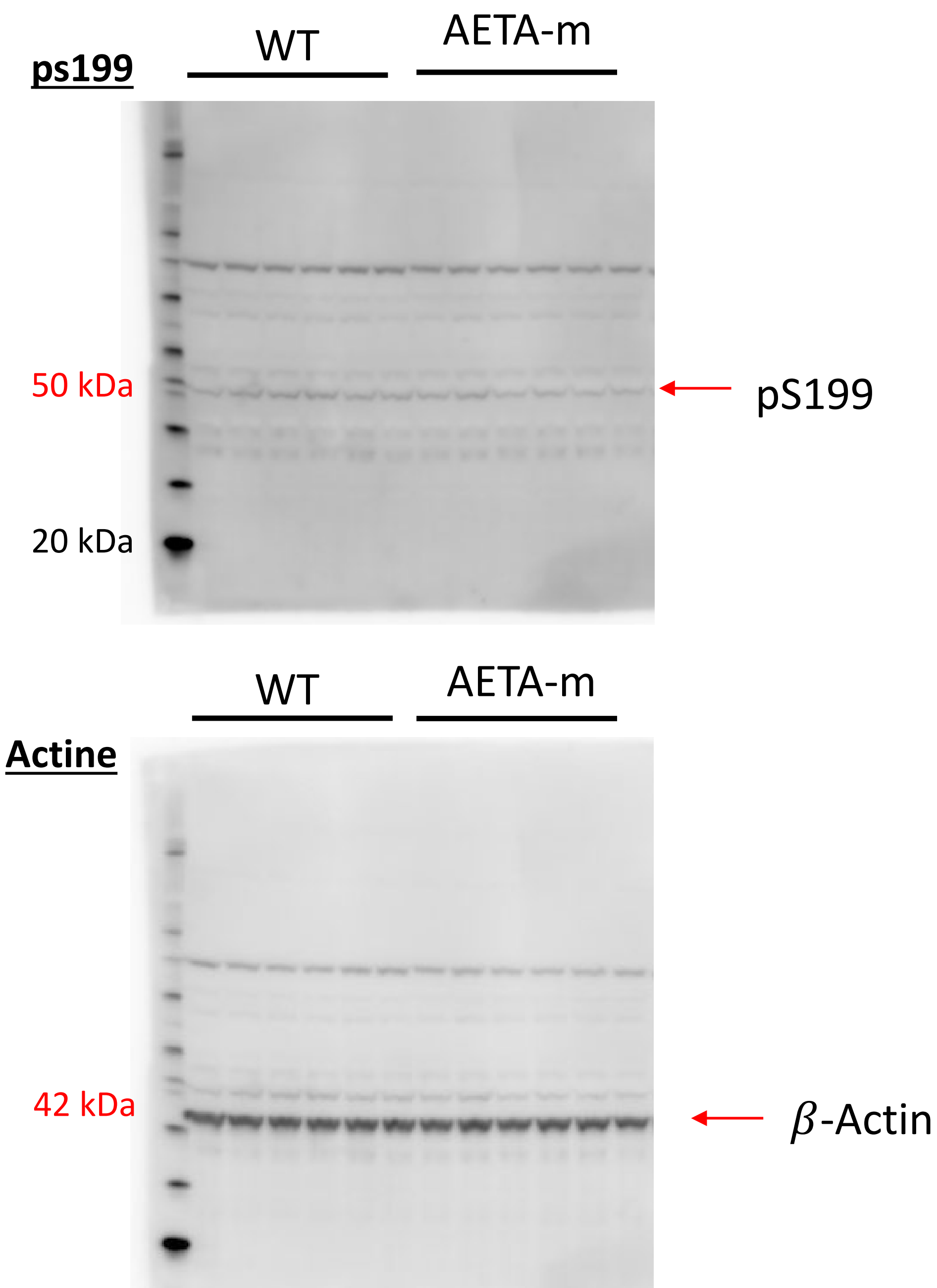

Figure S3d: pS396 in AETA-m mice

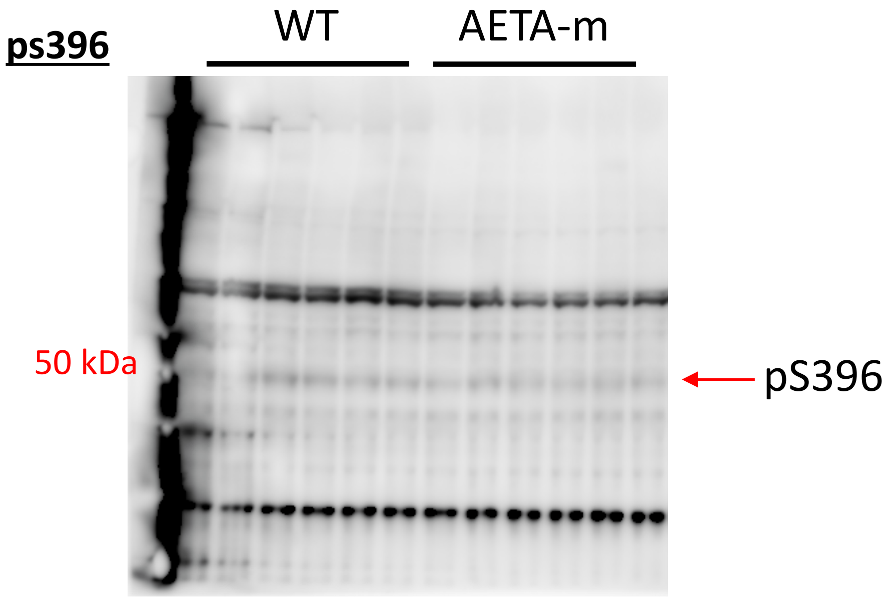

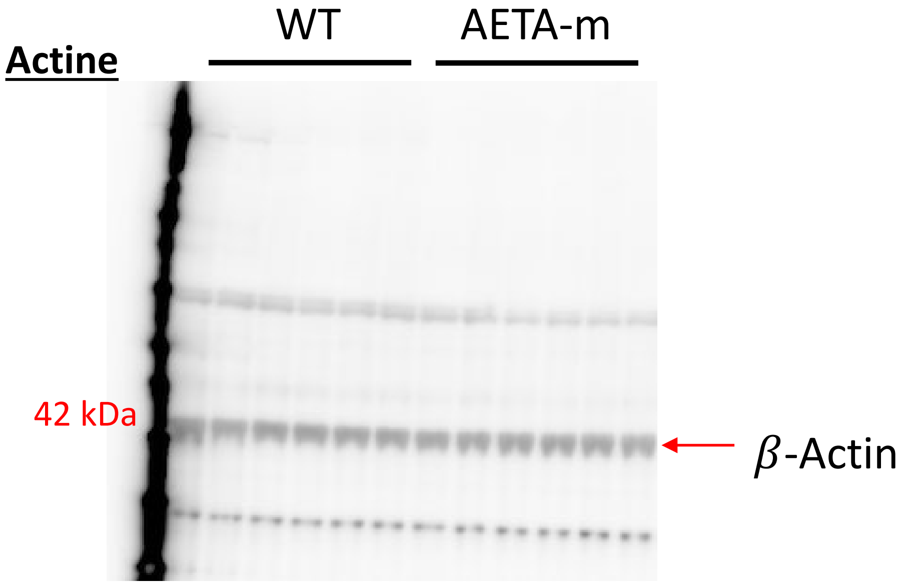

Figure 4e: p38 p-p38 in AETA-m female mice

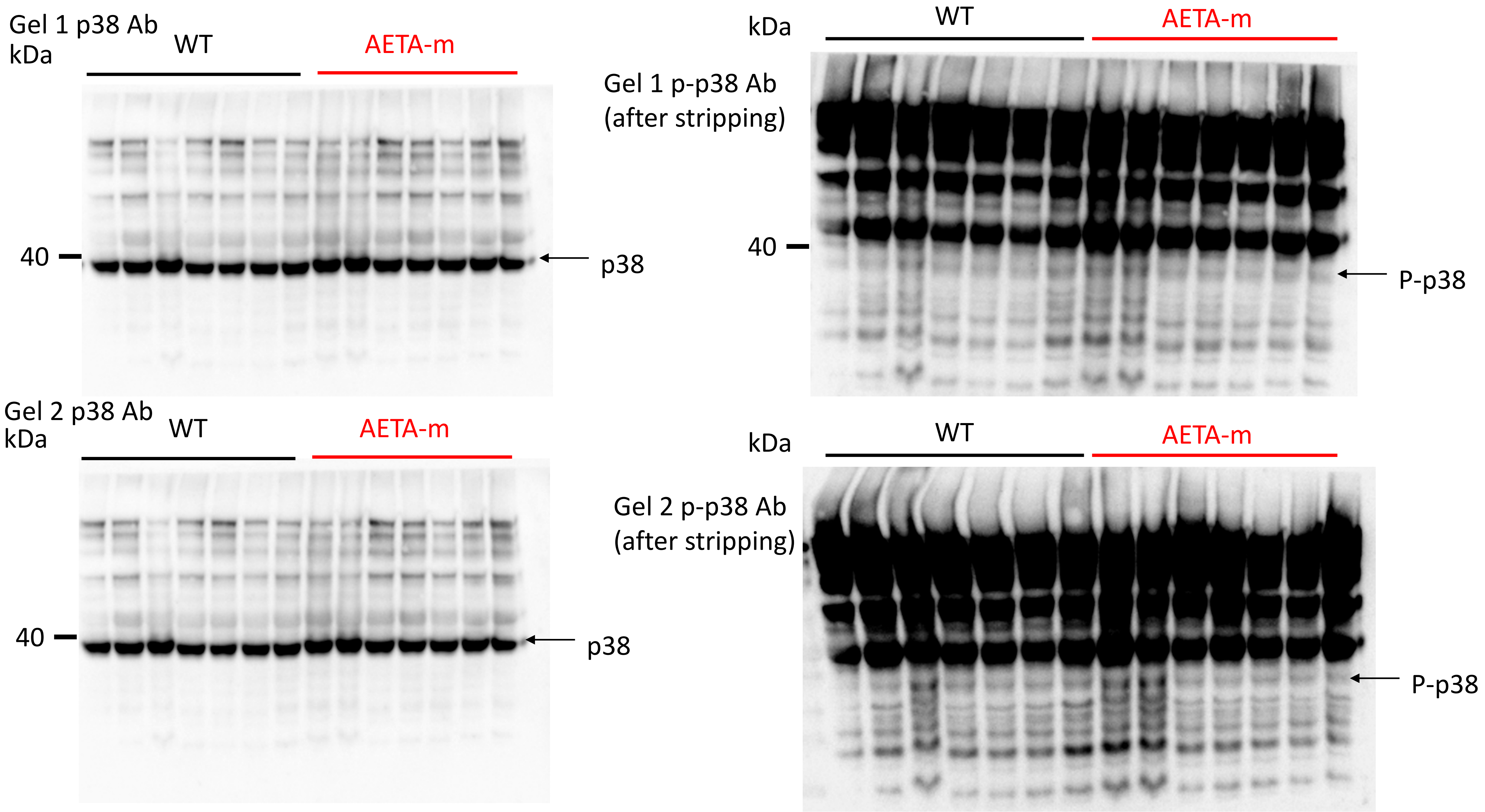

Figure 4e: p38 p-p38 in AETA-m female mice

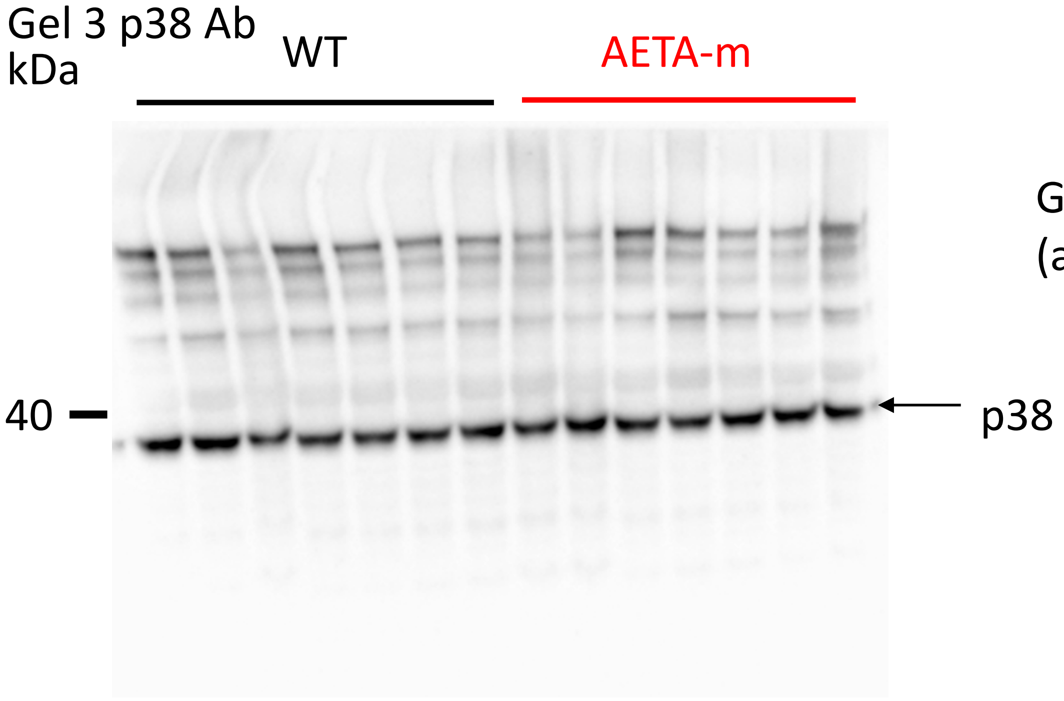

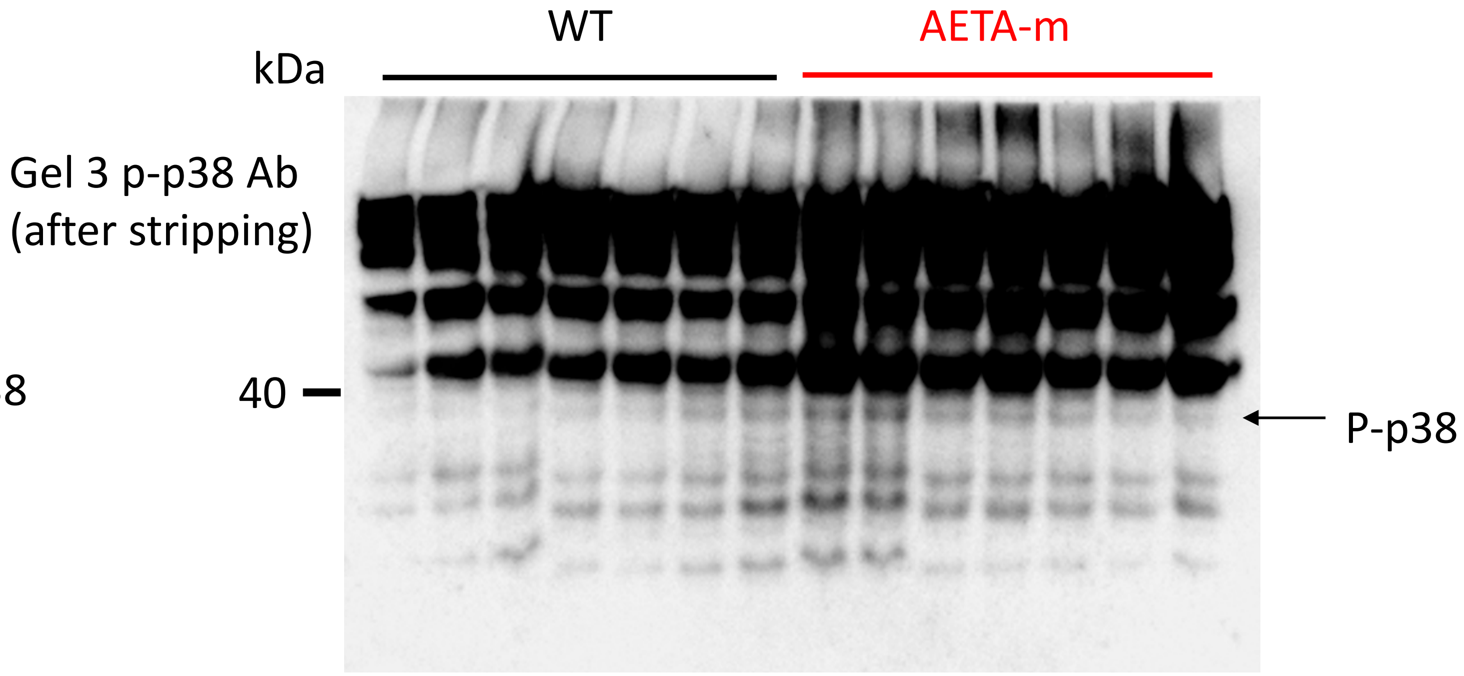

Figure S5e: p38 p-p38 in AETA-m male mice

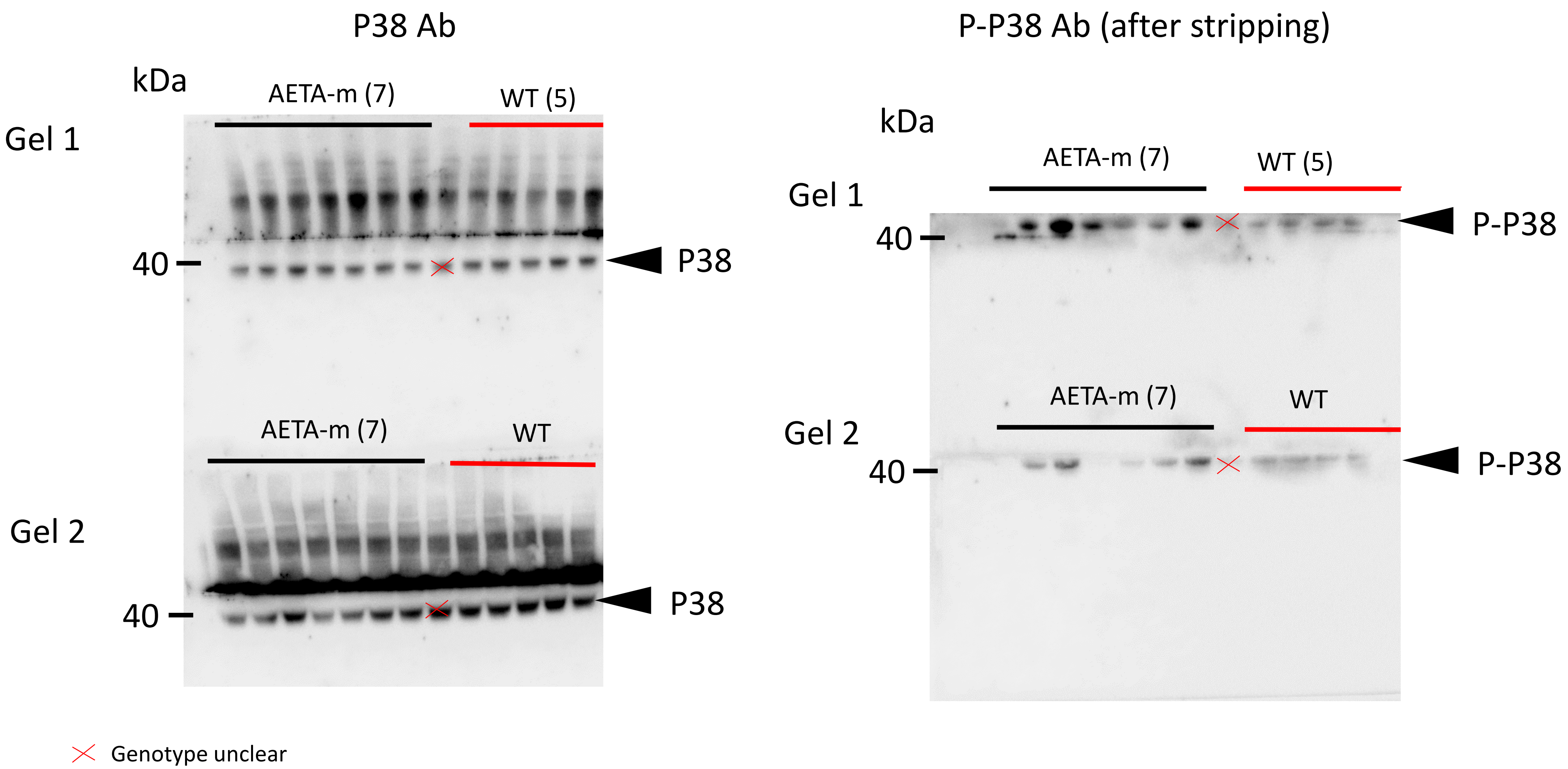

Figure S5e: p38 p-p38 in AETA-m mice

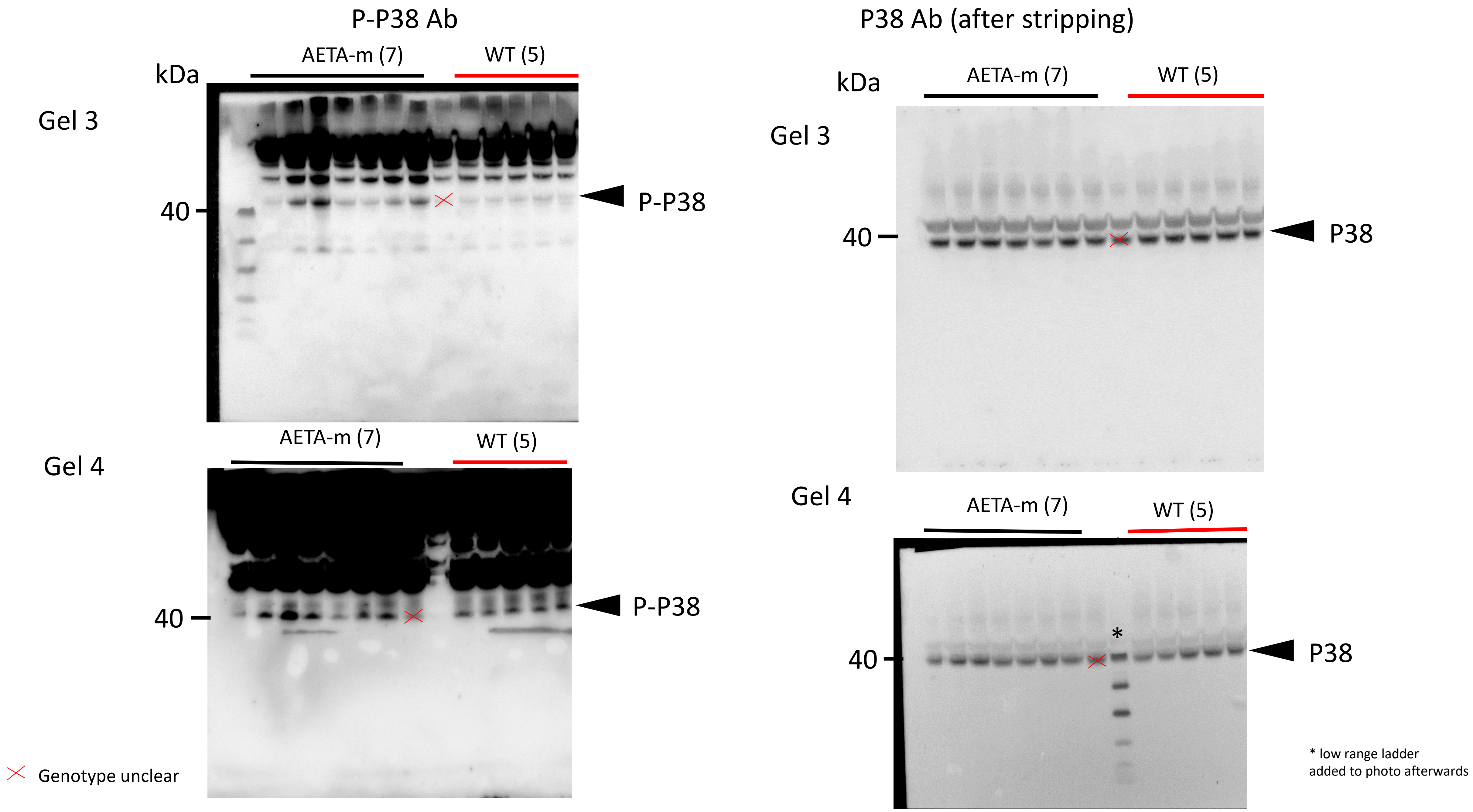

Supplement: Supplementary file 2 — Supplementary file2 (PDF 21615 KB) [file 401_2026_3033_MOESM2_ESM.pdf]
