# Supplementary material for: AETA peptide contributes to Alzheimer’s disease signature of synapse dysfunction
Source: Acta Neuropathol. 2026 Jun 3;151(1):65. doi: 10.1007/s00401-026-03033-2 (PMC13233929; doi:10.1007/s00401-026-03033-2)
Supplement: Supplementary file 3 — Supplementary file3 (PDF 2626 KB) [file 401_2026_3033_MOESM3_ESM.pdf]

Supplementary information

Title: AETA peptide contributes to Alzheimer's disease signature of synapse dysfunction.

Journal: Acta Neuropathologica

Jade Dunot[1*], Carine Gandin[1], Marin Truchi[1], Giulia Pirro[1], Sébastien Moreno[1], Agathe Launay[2], Benjamin Azoulay[1], Hugo Landra[1,3], Sandy Ma Yishan[1,3], Luc Buée[2], Kevin Lebrigand[1], Paula A. Pousinha[1], David Blum[2], Bernard Mari[1], Ingrid Bethus[1], Michael Willem[4] and Hélène Marie[1*].

[1] Université Côte d'Azur, CNRS, INSERM, Institut de Pharmacologie Moléculaire et Cellulaire, Valbonne, France ; [2] Univ. Lille, INSERM, CHU Lille, UMR-S1172 LilNCog - Lille Neuroscience & Cognition, Lille, France; [3]Munich Cluster for Systems Neurology (SyNergy), Munich, Germany; [4] Biomedical Center (BMC), Division of Metabolic Biochemistry, Faculty of Medicine, Ludwig-Maximilians-Universität München, Munich, Germany

* Corresponding authors : Hélène Marie & Jade Dunot

Email: marie@ipmc.cnrs.fr; jade@ad6d.net

Figures S1-S6

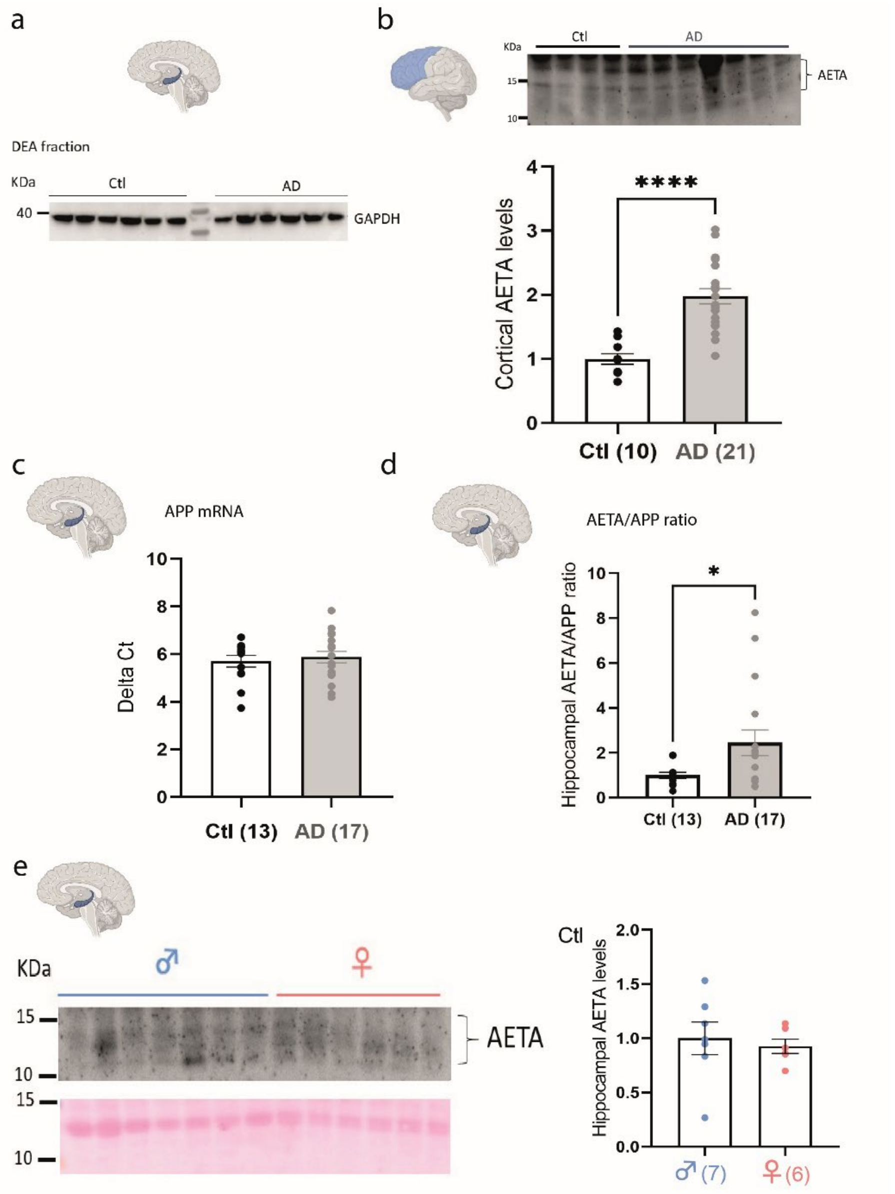

Figure S1 (related to Figure 1): Additional AETA and APP quantification in hippocampi of AD patients and quantification of AETA in prefrontal cortex (PFC) of AD patients. (a) General tissue integrity was confirmed by examples of GAPDH immunoblotting in DEA fractions of protein lysates. **(b)** Immunoblot (2D9 antibody) examples and quantification of AETA levels in PFC of AD patient vs control subjects (relative levels normalized to age-matched control (Ctl) subjects; normalized to actin loading control – see full blots used for analysis provided in Supplemental material). **(c)** Quantification of APP transcript by qPCR in hippocampi of human control subjects (Ctl) and AD brains. **(d)** The AETA/APP ratio was calculated from results obtained in graphs of Figure 1b-c. **(e)** Immunoblot (2D8 antibody and ponceau staining for normalization) examples (left) and quantification of AETA levels in hippocampi of male vs female control subjects (right). N= number of human samples; each sample was tested in triplicate and presented as mean. Error bars represent s.e.m.; * $p < 0.05$; **** $p < 0.0001$. Statistics: Welch's test for b and e, and Mann-Whitney test for c et d. See full statistics in Supplemental Table S9.

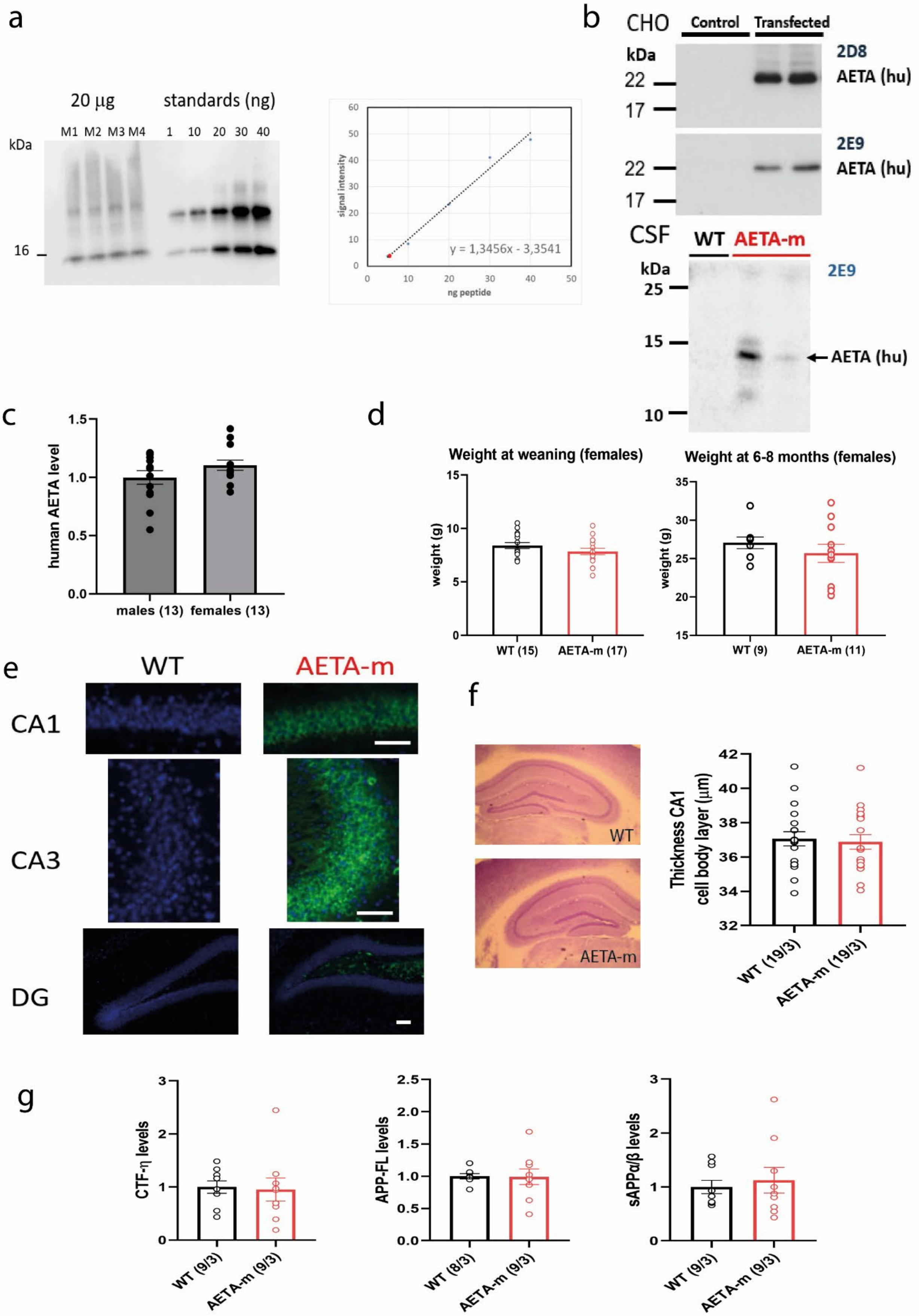
a
20 µg
standards (ng)
M1 M2 M3 M4
1 10 20 30 40
kDa
16
signal intensity
ng peptide
y = 1,3456x - 3,3541
b
CHO
Control
Transfected
kDa
2D8
AETA (hu)
2E9
AETA (hu)
CSF
WT
AETA-m
2E9
AETA (hu)
c
human AETA level
males (13)
females (13)
d
Weight at weaning (females)
Weight at 6-8 months (females)
weight (g)
WT (15)
AETA-m (17)
WT (9)
AETA-m (11)
e
WT
AETA-m
CA1
CA3
DG
f
WT
AETA-m
Thickness CA1 cell body layer (µm)
WT (19/3)
AETA-m (19/3)
g
CTF-η levels
APP-FL levels
sAPPα/β levels
WT (9/3)
AETA-m (9/3)
WT (8/3)

Figure S2 (related to Figure 2): Additional characterization of AETA-m mice overexpressing secreted human AETA in the brain. (a) (left) Immunoblot for quantification (2E9 antibody) of recombinant human AETA in hippocampi of four male AETA-m mice (6 months old; M1-M4). The quantification was made against a standard curve of synthetic AETA (1, 10, 20, 30, 40 ng). Note that recombinant AETA seems to partially dimerize both in brain and *in vitro*. (Right) Both bands of the synthetic peptide were used to generate the standard curve and calculate the average AETA quantity using the equation shown in graph (ng/μg protein; red dot on graph represents average quantity of human AETA calculated as 4,07 signal intensity in 20 μg of extracted protein; which gives 0,27 ng/μg using the provided equation and divided by 20 to obtain per quantity per μg). **(b)** Confirmation of secretion of recombinant human AETA: Detection of secreted human AETA-mycflag in medium of CHO transfected cells (AETA) and not in untransfected cells (control) by immunoblotting with 2D8 and 2E9 (top). Detection of human AETA in CSF of female AETA-m mice and not of WT mice by immunoblotting with 2E9 (bottom). **(c)** Quantification of human AETA levels (2E9 antibody on DEA lysate) in hippocampi of 6-8 months old female and male AETA-m mice. **(d)** Weight of female WT and AETA-m mice at weaning (21 days after birth) and at 6-8 months of age. **(e)** Representative examples of staining of human AETA peptide with 2E9 in CA1, CA3 cell body layers, and DG showing stronger expression within the CA layers in AETA-m mice compared to WT mice. . Nuclei were stained with DAPI (blue). Scale bars represent 50 μm. **(f)** Example of cresyl violet staining of hippocampi of male WT and AETA-m mice and measure of CA1 cell body layer thickness in these slices. **(g)** Quantification of endogenous levels of CTF-η, full-length APP (APP-FL) and sAPP α/β in WT and AETA-m mouse brains by immunoblotting. Full blots used for analysis are provided in Supplemental material. Three mouse brains per genotype were processed in triplicates. n= mice for c-d; n/N= slices/ mice for f; n/N= replicates/ mice for g. Error bars represent s.e.m. Statistics: Mann-Whitney test for c,d g left and e right. Student t-test for f and g center. See full statistics in Supplemental Table S11.

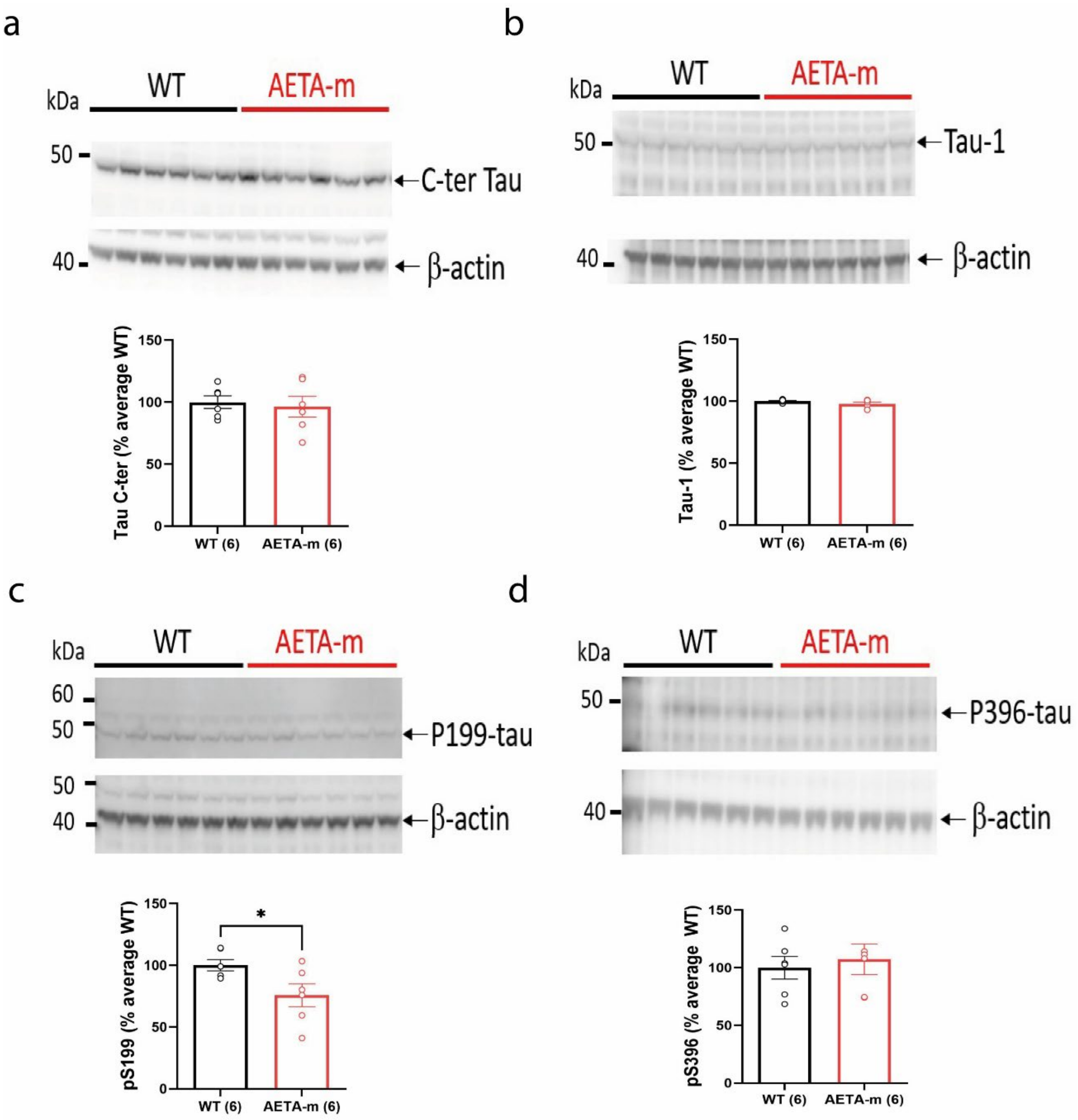

Figure S3 -Tau levels and Tau phosphorylation do not increase in hippocampi of adult female AETA-m mice. Immunoblots and associated quantification of endogenous levels of tau with C-terminal (a) and Tau-1 (b) antibodies and of endogenous tau phosphorylation at positions (c) S199 and (d) S396 in 6-8 months old female mice. Full blots used for analysis are provided in Supplemental material. n= mice. Error bars represent s.e.m.; * $p < 0.01$. Statistics: Student t-test. See full statistics in Supplemental Table S12.

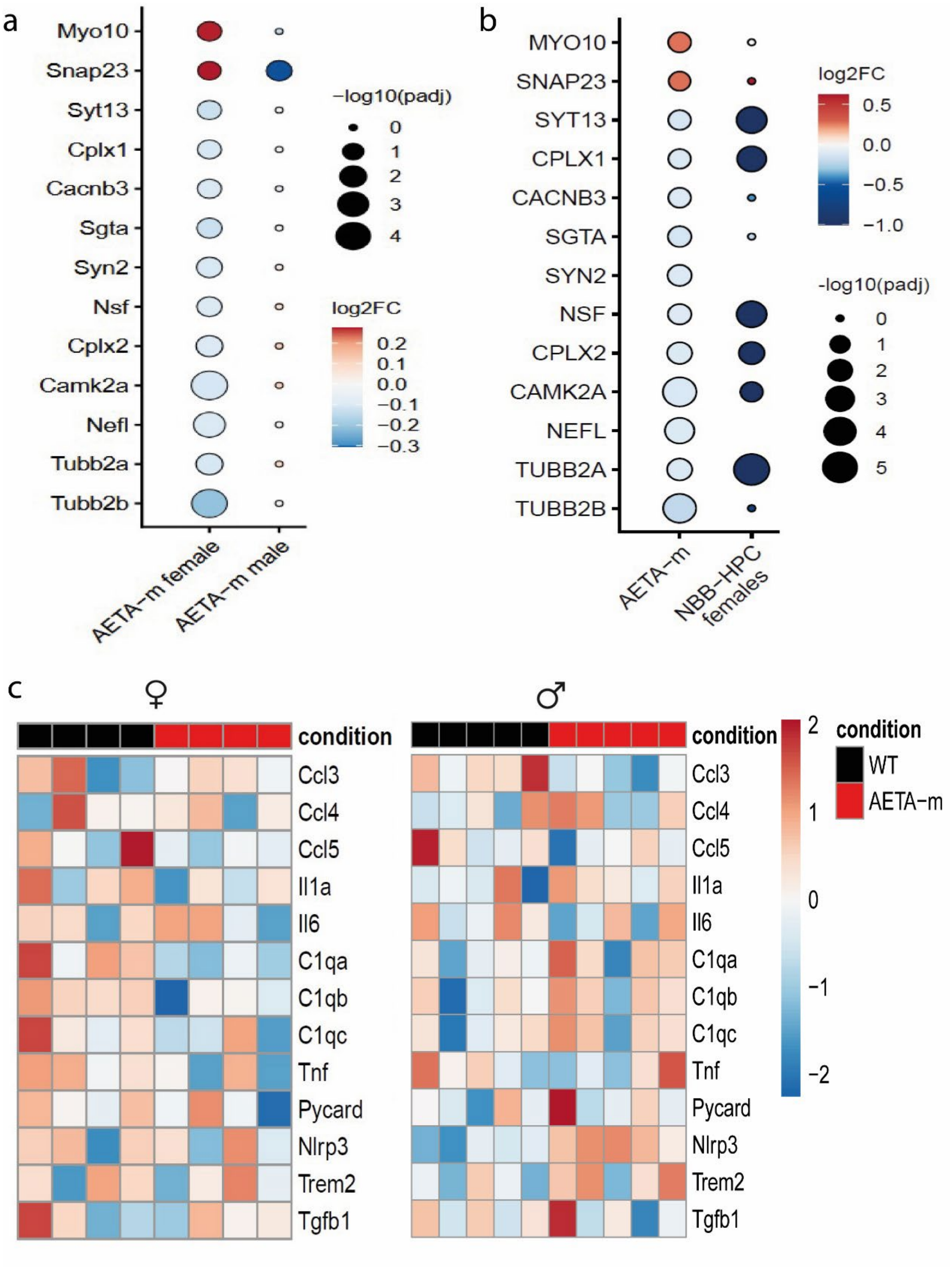

Figure S4 (related to Figure 2 and 3) Additional analysis of bulk RNA sequencing and comparison to human NBB-HPC database. (a) Lack of molecular signature of synaptic genes pertinent to AD in male AETA-m hippocampi. **(b)** Comparison of synaptic molecular signature between female AETA-m hippocampi and female-only NBB-HPC data. **(c)** Levels of mRNAs pertaining to neuroinflammation-related genes were unchanged in female (left) and male (right) hippocampi of AETA-m mice. For (c), see full statistics in Supplemental Tables S4 and S7.

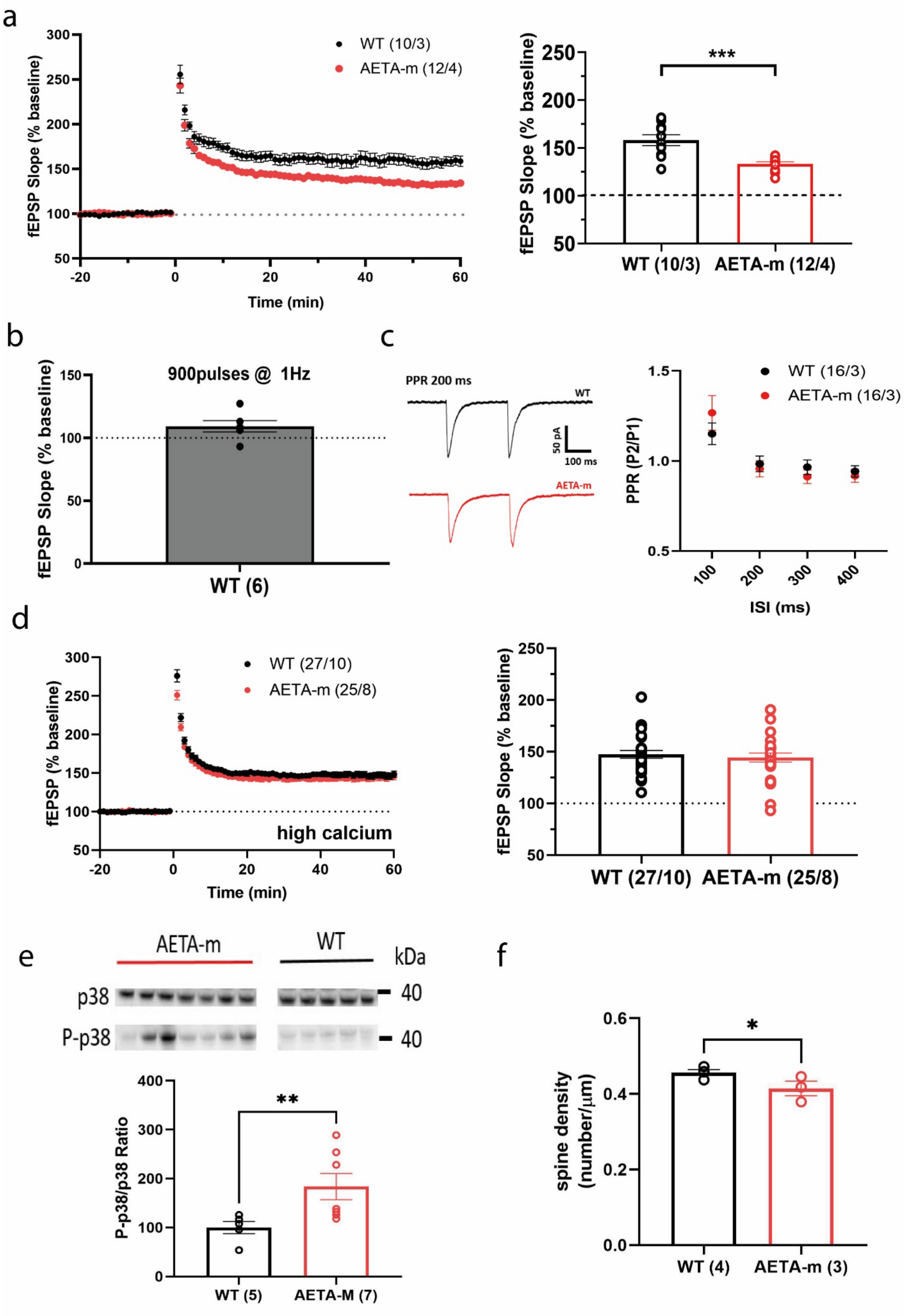

a
WT (10/3)
AETA-m (12/4)
fEPSP Slope (% baseline)
Time (min)
***
WT (10/3)
AETA-m (12/4)
b
900pulses @ 1Hz
fEPSP Slope (% baseline)
WT (6)
c
PPR 200 ms
WT
AETA-m
50 pA
100 ms
WT (16/3)
AETA-m (16/3)
PPR (P2/P1)
ISI (ms)
d
WT (27/10)
AETA-m (25/8)
fEPSP (% baseline)
high calcium
Time (min)
fEPSP Slope (% baseline)
WT (27/10)
AETA-m (25/8)
e
AETA-m
WT
kDa
p38
P-p38
40
40
P-p38/p38 Ratio
**
WT (5)
AETA-M (7)
f
*
spine density (number/μm)
WT (4)
AETA-m (3)

Figure S5 (related to Figure 4): Additional data of synapse function, plasticity and spine density in CA1 pyramidal neurons of AETA-m mice. (a) Time course (left; LTP induction at time 0) and bar graph (right, 45-60 min post-induction) of long-term potentiation of fEPSP slope (% baseline) at CA3-CA1 synapse in hippocampal slices of male WT and AETA-m mice. **(b)** LTD could not be induced in adult female WT mice. **(c)** Representative traces (left) and paired pulse ratio (pulse 2/pulse 1) analysis (right) measured in CA1 pyramidal neurons of female WT and AETA-m mice. **(d)** Time course (left, LTP induction at time 0) and bar graph (right, 45-60 min post-induction) of long-term potentiation of fEPSP slope (% baseline) at CA3-CA1 synapse in hippocampal slices of male WT and AETA-m mice recorded with aCSF containing high calcium. **(e)** Example immunoblots of p38 and phosphorylated p38 (P-p38) and P-p38/p38 ratio quantification in male WT and AETA-m mice. Samples were tested in quadruplicate. **(f)** Spine density measured on dendrites of the stratum radiatum of CA1 pyramidal neurons of male WT and AETA-m mice. Error bars represent s.e.m.; * $p < 0.05$; ** $p < 0.01$; *** $p < 0.001$. n/N= slices/mice for a and d; n/N= cells/mice for c; n= number of mice for e-f. Statistics : Student t-test for a and d; Two-way ANOVA for c; Mann-Whitney for e and f. See full statistics in Supplemental Table S14.

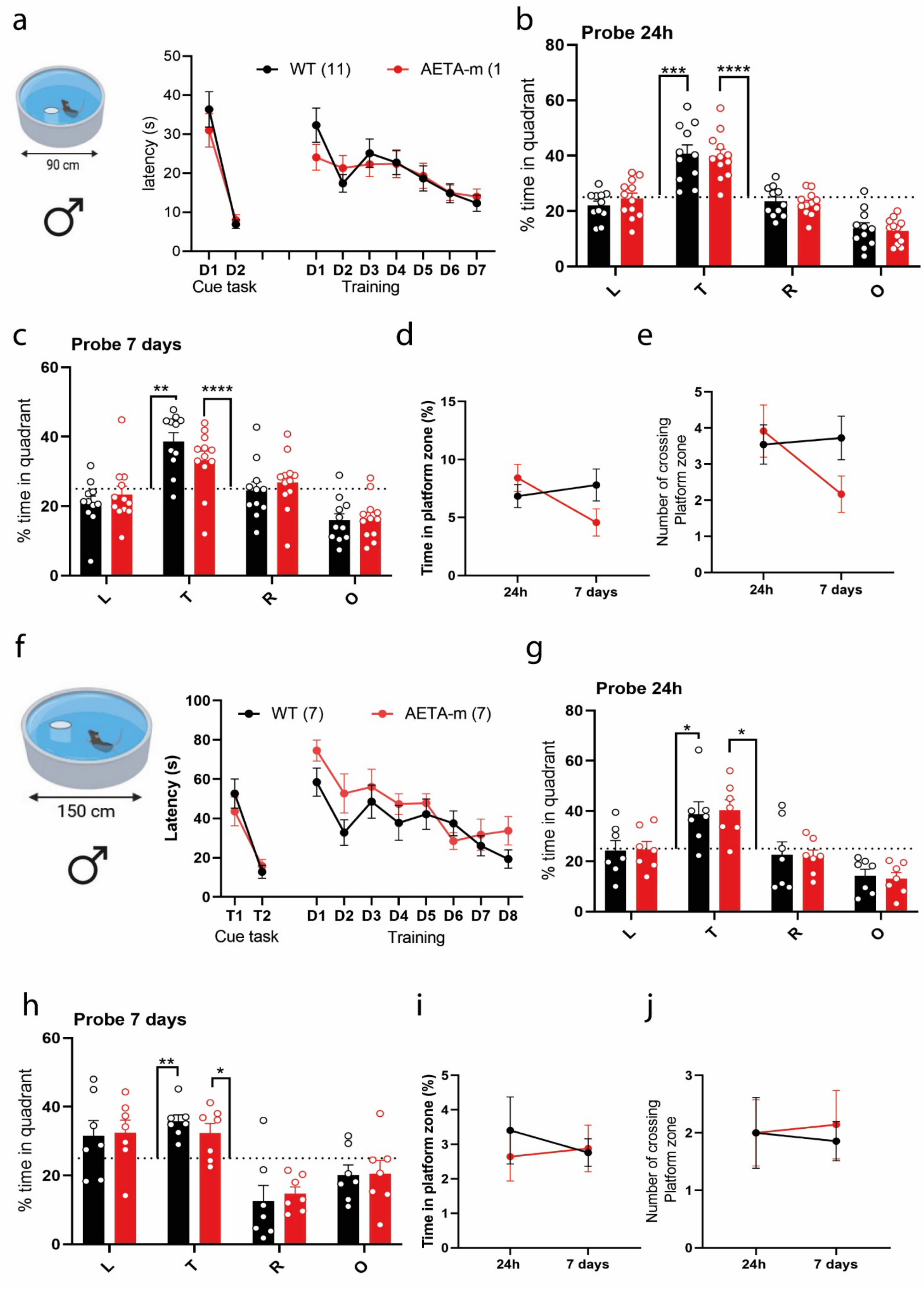

a
90 cm
latency (s)
WT (11)
AETA-m (1
D1 D2
Cue task
D1 D2 D3 D4 D5 D6 D7
Training
b
Probe 24h
% time in quadrant
***
****
L T R O
c
Probe 7 days
% time in quadrant
**
****
L T R O
d
Time in platform zone (%)
24h 7 days
e
Number of crossing Platform zone
24h 7 days
f
150 cm
Latency (s)
WT (7)
AETA-m (7)
T1 T2
Cue task
D1 D2 D3 D4 D5 D6 D7 D8
Training
g
Probe 24h
% time in quadrant
*
*
L T R O
h
Probe 7 days
% time in quadrant
**
*
L T R O
i
Time in platform zone (%)
24h 7 days
j
Number of crossing Platform zone
24h 7 days

Figure S6 (related to Figure 5): Long-term memory in male AETA-m mice. (a) Latency (s) during cue task and training in 90 cm Morris water maze pool. **(b-c)** % time spend in quadrants (T: target, L: left, R: right, O: opposite) during probe test (b) at 24h, (c) at 7 days. **(d)** Time spent in platform zone at 24h and 7 days. **(e)** Number of platform crossing at 24h and 7 days. **(f)** Latency (s) during cue task and training in 150 cm Morris water maze pool. **(g-h)** % time spend in quadrants during probe test at 24h and at 7 days. **(i)** Time spent in platform zone at 24h and 7 days. **(j)** Number of platform crossing at 24h and 7 days. N= mice. Error bars represent s.e.m.; *p < 0.01; ** p < 0.001; *** p < 0.0001; **** p < 0.0001; against chance level (25%, dotted line on graph). Statistics: two-way ANOVA. See full statistics in Supplemental Table S16.
